# Effect of Ca^2+^ Doping on the Structural, Magnetic and Magneto–Transport Properties of La_1−*x*_Ca*_x_*MnO_3_ Manganites: Insights from XPS and ^55^Mn IFNMR Studies

**DOI:** 10.3390/molecules31173122

**Published:** 2026-09-06

**Authors:** Arun Kumar Shavi Mallikarjuna, Manjunatha Mushtagatte, Gavinolla Srinivas Reddy, Selvaraj Anandh Jesuraj, Mangesh Lodhe, David Laroze, Thipperudrappa Javuku

**Affiliations:** 1 Department of Studies in Physics, Vijayanagara Sri Krishnadevaraya University, Ballari 583105, Karnataka, India; arunkingo123@gmail.com; 2 Department of Physics, SGVVT’s SG College, Koppal 583231, Karnataka, India; 3 Department of Physics, School of Engineering, Presidency University, Yelahanka, Bangalore 560064, Karnataka, India; 4 Centre for Nanoscience and Nanotechnology, Sathyabama Institute of Science and Technology, Chennai 600119, Tamil Nadu, India; 5 Department of Materials and Metallurgical Engineering, Maulana Azad National Institute of Technology, Bhopal 462003, Madhya Pradesh, India; 6 Instituto de Alta Investigación, Universidad de Tarapacá, Casilla 7D, Arica 1000000, Chile

**Keywords:** magnetoresistance, double-exchange interaction, spin polarized tunneling, variable-range hopping, ^55^Mn IFNMR

## Abstract

In this study, La_1−*x*_Ca*_x_*MnO_3_ (LCMO) samples (*x* = 0.3, 0.4 and 0.5) were synthesized using a sol–gel method and their structural, electronic, magnetic and magneto–transport properties were investigated. Structural analysis by XRD confirmed orthorhombic perovskite structure. FTIR studies indicated the changes in bond length and bond angle of Mn–O/Mn–O–Mn bonds; FESEM/EDAX confirmed a polycrystalline nature. Magneto–transport studies revealed a decrease in ferromagnetic metal–paramagnetic semiconductor transition temperature with the increase in Ca^2+^ content. Magneto–transport studies also revealed that the resistivity in the ferromagnetic metallic region was predominantly governed by the extrinsic spin–polarized tunneling (SPT) mechanism along with double-exchange interaction, whereas charge transport in the paramagnetic semiconducting region at higher temperatures was due to variable-range hopping conduction. Magnetoresistance was at its maximum near the transition temperature, but decreased at lower temperature (77 K). XPS and ^55^Mn IFNMR confirmed coexistence of Mn^3+^/Mn^4+^, while IFNMR results at 77 K confirmed high-frequency electron exchange among Mn^3+^ and Mn^4+^ ions due to double exchange interaction. VSM studies at 300 K showed that the paramagnetic nature of the samples and susceptibility decreased with the increase in Ca^2+^ content. The close agreement between the magnetoresistance behavior and the ^55^Mn IFNMR results confirms the role of the intrinsic DE interaction in governing the magnetotransport properties of LCMO.

## 1. Introduction

Rare-earth manganites with the general formula R_1−*x*_A*_x_*MnO_3_, where R is a trivalent rare-earth ion and (A) is a divalent alkaline earth ion, have attracted considerable interest because of the strong coupling among charge, spin, orbital and lattice degrees of freedom, giving rise to remarkable physical phenomena such as magnetoresistance (MR), charge/orbital ordering and electronic phase separation [1,2,3,4]. In these materials, the MR effect originates primarily from the magnetic field induced alignment of Mn spins, which suppresses spin-dependent scattering and enhances electron hopping through the double-exchange (DE) interaction [5,6]. The DE interaction is strongly influenced by structural distortions, particularly the Jahn–Teller distortion of MnO_6_ octahedra, electron–phonon coupling and chemical substitution at the A–site, all of which modify the electronic bandwidth and magnetic exchange interactions [6,7]. Consequently, the charge transport and magnetic properties of manganites can be effectively tuned by dopant concentration, oxygen stoichiometry, lattice strain and external parameters such as temperature, pressure and magnetic field [8,9,10].

Among the manganite family, La_1−*x*_Ca*_x_*MnO_3_ (LCMO) has attracted considerable research attention because its electronic and magnetic properties evolve systematically with Ca^2+^ concentration. The parent compound (LaMnO_3_) is an antiferromagnetic insulator, whereas substitution of Ca^2+^ for La^3+^ converts it to a ferromagnetic metallic state. This is due to the introduction of holes by Ca^2+^ doping, leading to the conversion of corresponding fraction of Mn^3+^ ions into Mn^4+^ ions to maintain charge neutrality, which promotes double-exchange (DE) interaction [11,12]. In addition to the DE interaction, the physical properties of manganites are strongly influenced by the Jahn–Teller (JT) distortion associated with the Mn^3+^ ions. Y. Tokura [12] reported that charge transport in manganites occurs through the hopping of Mn 3d (*e_g_*) electrons between Mn^3+^ and Mn^4+^ ions, a process that is significantly affected by electron–electron interactions and the Jahn–Teller effect. The local Jahn–Teller distortion, caused by the displacement of oxygen ions surrounding the Mn sites, plays an important role in governing both the metal–insulator transition and the ferromagnetic–paramagnetic phase transition in these materials [12].

The transport behaviour of LCMO also depends on its microstructure. Single crystals provide information on intrinsic transport but usually exhibit significant magnetoresistance only at relatively low temperatures [13]. Thin films and multilayer heterostructures offer opportunities for tailoring interface related transport phenomena; however, their properties are strongly influenced by epitaxial strain, substrate effects and fabrication conditions [14,15]. Polycrystalline ceramics are comparatively easier to synthesize and are well suited for investigating both intrinsic magnetic interactions and extrinsic contributions arising from grain boundaries, which can significantly influence low-temperature magnetotransport [3,16].

Previous studies have established several important aspects of magnetotransport in polycrystalline manganites. H Felhi et al. [17] showed that, among several proposed models, the spin-polarized tunneling (SPT) model provides the best description of the low–temperature resistivity by considering SPT between neighbouring ferromagnetic grains, whereas other mechanisms such as Coulomb blockade and electron–electron/electron–magnon scattering fail to reproduce the experimental behavior satisfactorily. M. I. Auslender et al. [18] reported that the SPT model provides an effective description of the low-temperature resistivity in polycrystalline samples by considering spin-dependent electron tunneling between neighbouring ferromagnetic grains across grain boundaries. Hundley et al. [19] and Martinez et al. [20] showed that polaron hopping contributes to charge transport above the Curie temperature. This contribution is progressively suppressed below Tc. Gharsallah et al. [21] reported a crossover from electron–electron and electron–magnon scattering at low temperatures to thermally activated small-polaron hopping at high temperatures. More recently, Jithin et al. [22] correlated the evolution of the Griffiths phase with enhanced MR in half-doped manganites (La_0.5_Ca_0.5_MnO_3_). In addition, ^55^Mn internal field nuclear magnetic resonance (IFNMR) has proved to be a sensitive local probe of the magnetic environment and has been used to measure the hyperfine field at the Mn nuclei, which reflects the local magnetic moment, magnetic exchange interactions among Mn ions, and intrinsic double-exchange interaction [23,24,25,26,27].

In the present work, polycrystalline La_1−*x*_Ca*_x_*MnO_3_ (LCMO) (*x* = 0.3, 0.4 and 0.5) samples were synthesized using the sol–gel method. The structural, morphological, magnetic and magnetotransport properties of the synthesized samples were investigated using X–ray diffraction (XRD), Fourier transform infrared spectroscopy (FTIR), field emission scanning electron microscopy with energy dispersive X–ray spectroscopy (FESEM/EDX), X–ray photoelectron spectroscopy (XPS), ^55^Mn IFNMR, magnetic measurements and magnetotransport studies. The objective was to correlate structural characteristics, Mn valence states, local magnetic information from ^55^Mn IFNMR and the corresponding magnetic and electrical transport properties to Ca^2+^ concentration. To the best of our knowledge, the present study is the first to report analysis of the DE mechanism in LCMO samples using the ^55^Mn IFNMR technique. The IFNMR signal in the range of 380 MHz to 388 MHz provided direct experimental evidence of DE interaction mechanism. In the present study, correlation between the magnetotransport properties and the ^55^Mn IFNMR results is established to demonstrate the association between MR and intrinsic double-exchange interaction.

## 2. Results and Discussion

### 2.1. Structural and Morphological Studies

#### 2.1.1. X-Ray Diffraction (XRD) Studies

**Figure 1:**
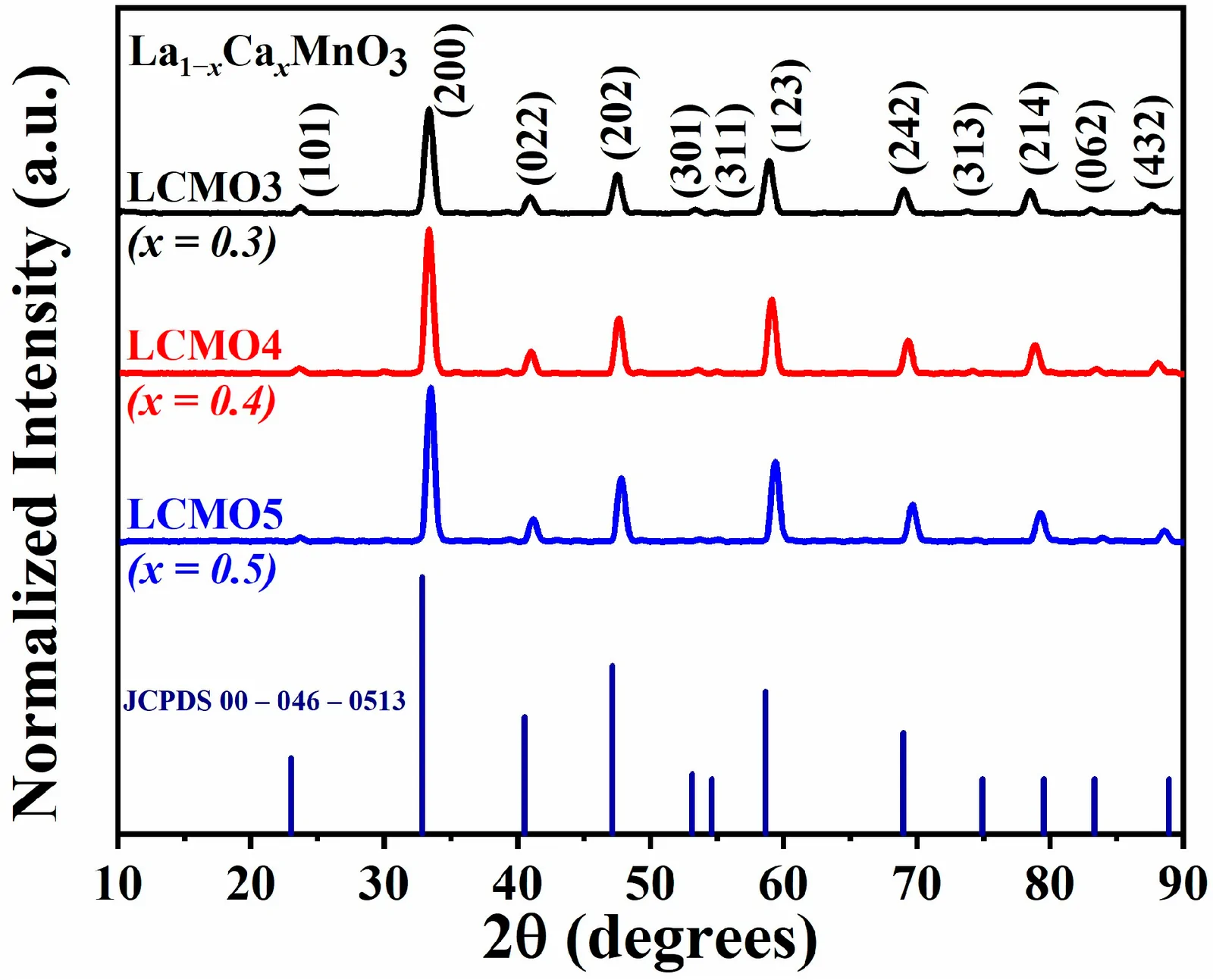
Room-temperature XRD patterns of La_1−*x*_Ca*_x_*MnO_3_ samples LCMO3, LCMO4 and LCMO5 (*x* = 0.3, 0.4 and 0.5) compared with the standard JCPDS reference pattern.

The XRD spectrum of the synthesized La_1−*x*_Ca*_x_*MnO_3_ samples was recorded at room temperature and analysed using Rietveld refinement in order to identify the crystal structure and space group, as well as to estimate the structural and lattice parameters. Figure 1 represents the room-temperature XRD patterns of LCMO3, LCMO4 and LCMO5 samples together with the corresponding reference JCPDS card 00–046–0513.

The diffraction peaks appearing at 2θ values of ~24, ~33, ~41, ~48, ~53, ~54, ~59, ~69, ~75, ~79, ~83 and ~89 correspond to the (101), (200), (022), (202), (301), (311), (123), (242), (313), (214), (062) and (432) crystallographic (*hkl*) planes, respectively. All the observed XRD peaks are well indexed to an orthorhombic perovskite lattice, with the Pnma–62 space group consistent with standard reference pattern and previous reports [28,29,30]. From Figure 1, it is also evident that the peak intensity increases and the peaks become narrower as the Ca^2+^ content in the LCMO samples increases. These variations in the XRD peaks reflect changes in lattice parameters and microstructural features such as crystallite size and lattice strain. To obtain accurate lattice parameters, Rietveld refinement was performed using the *FullProf* program (ver. 8.40). The Rietveld refinement converged successfully with R_p_, R_exp_ < 5% and R_wp_ < 10% (Table 1). These refinement indices are comparable to those reported in the literature [30,31]. The obtained goodness-of-fit values (*χ*^2^ = 1.465–1.864) confirm the reliability of the refined structural parameters. The Rietveld refined graphs of all the prepared LCMO samples are given in Figure S1 (Supplementary Materials). The extracted structural parameters from refinement are provided in Table 1 along with standard deviations. The uncertainty percentage of standard deviations are less than 0.1%. Table 1 shows that the unit cell parameters and cell volume contracted with the increase of Ca^2+^ substitution in the LCMO samples. This reduction in unit cell parameters and cell volume occurred due to the increase in smaller ionic radius Mn^4+^ concentration accompanied by a corresponding decrease in larger ionic radius Mn^3+^ ions due to the substitution of Ca^2+^ for La^3+^ [32]. The refined ideal perovskite lattice parameter (*a_p,ref_*) [33] was calculated using Equation (1), where V represents the unit cell volume obtained from the refinement. The theoretical ideal perovskite lattice parameter (*a_p,the_*) was also estimated from the ionic radii of the constituents in the LCMO samples, using Equation (2) [34]. In this equation, *x*, $r_{B}$ and $r_{O^{2-}}$ represent the cation doping concentration, radii of the B–site cation and the oxygen anion, respectively. These values are also provided in Table 1. The perovskite structure of LCMO is influenced by two factors: (a) MnO_6_ octahedral tilting and (b) Jahn–Teller (JT) distortions of Mn^3+^ ions [33]. From Table 1, it can be observed that the refined ideal perovskite lattice parameter is less than the theoretical ideal perovskite lattice parameter for all the LCMO samples. This suggests the presence of compressive strain along with significant MnO_6_ octahedral tilting and Jahn–Teller distortion. The difference between the refined and theoretical ideal perovskite lattice parameter (Δ*a_p_*) is also presented in Table 1. Table 1 reveals that as the Ca^2+^ concentration increases, the difference between the refined and theoretical ideal perovskite lattice parameter (Δ*a_p_*) decreases. This is attributed to the weakening of both MnO_6_ octahedral tilting and the Jahn–Teller distortion of the Mn^3+^ ions [35,36] with the increase in Ca^2+^ content.

(1) $$a_{p,ref}=\frac{V^{\frac{1}{3}}}{2}$$

(2) $$a_{p,the}=2(\langle r_{B}\rangle +r_{O^{2-}}),\;\mathrm{where}\;\langle r_{B}\rangle =(1-x).r_{Mn^{3+}}+(x).r_{Mn^{4+}}$$

**Figure 2:**
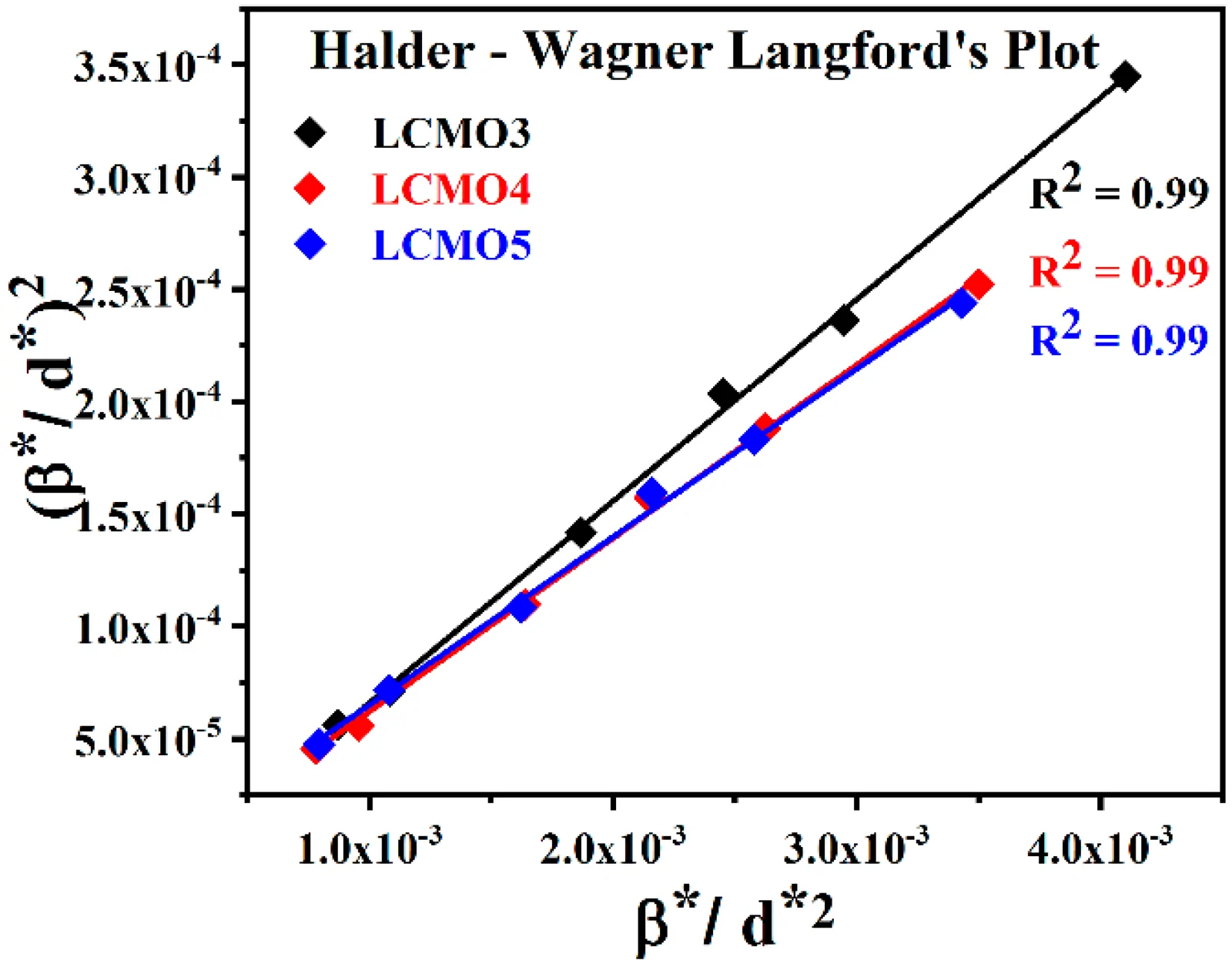
The HWL graphs of the LCMO3, LCMO4 and LCMO5 samples.

The crystallite size and lattice strain of the LCMO samples were determined using the well-established Halder–Wagner–Langford (HWL) method [26,27]. The details of the HWL method are reported in our previous studies [26,27]. The HWL plots of the LCMO samples are shown in Figure 2. The high R^2^ values of the HWL plots indicate the goodness of fit, authenticating the correctness of the estimated parameters. The crystallite size and lattice strain obtained from these plots are given in Table 2. Furthermore, the dislocation density (*δ*) was calculated using Equation (3) [37] and these values are also included in Table 2.

(3) $$\delta =\frac{1}{D^{2}}$$

The crystallite size estimated from the HWL analysis was 11–14 nm and the crystallite size increased with the increase in Ca^2+^ concentration. As the Ca^2+^ content increased, the melting point slightly decreased, providing effective atomic diffusion and grain boundary mobility leading to an increase in crystallite size. Furthermore, the decreased Jahn–Teller distortion with the increase in Ca^2+^ content aided crystal growth due to rearrangement of atoms into larger and cleaner domains. From Table 2, it is also clear that lattice strain and dislocation density decreased as the Ca^2+^ content increased. The decrease in lattice strain and dislocation density may have been due to the reduced distortion with the increase in Ca^2+^ content.

#### 2.1.2. Fourier Transform Infrared (FTIR) Spectroscopy Studies

**Figure 3:**
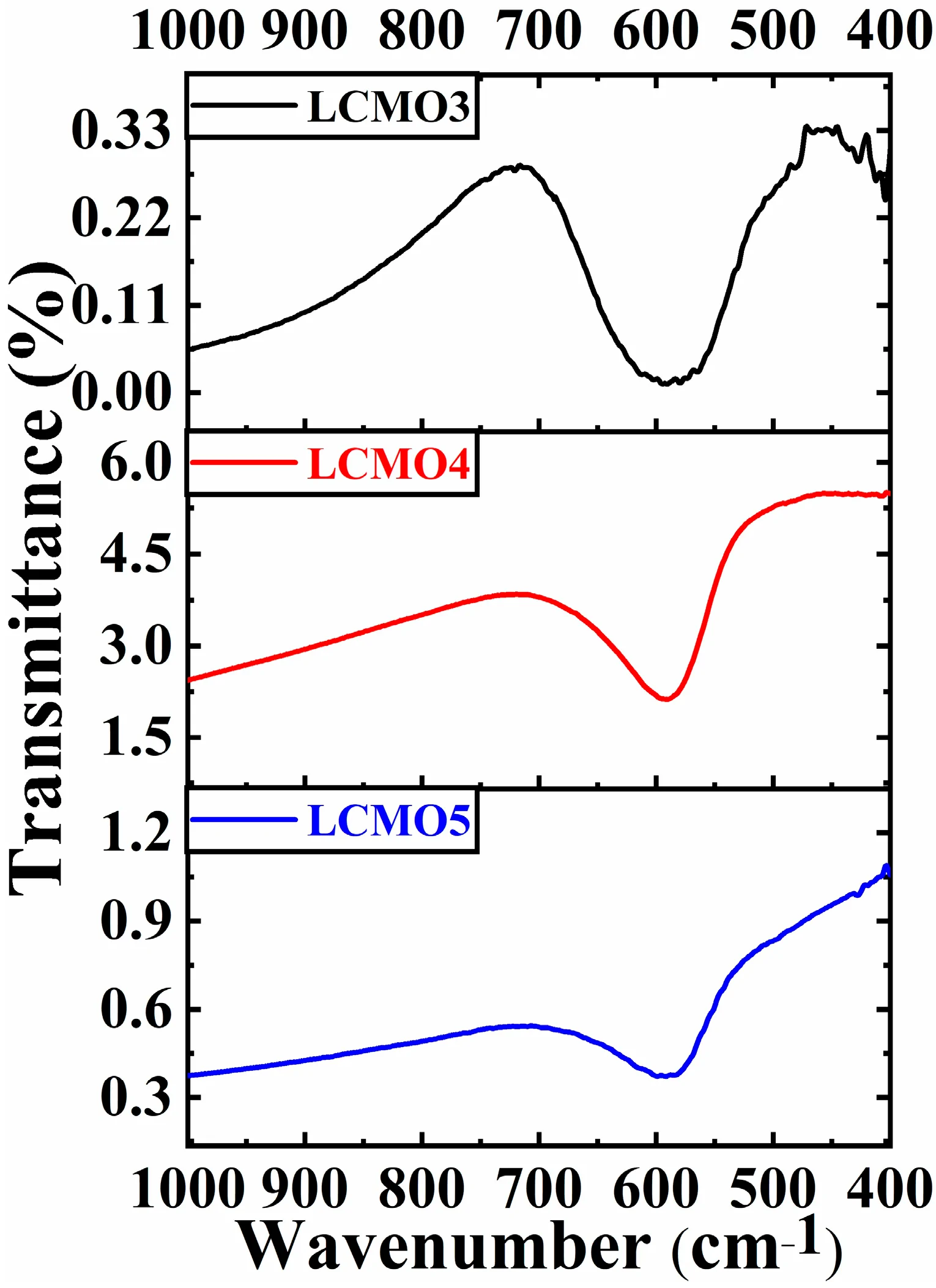
Room–temperature FTIR spectra of the synthesized LCMO samples recorded in the wavenumber range of 400 cm^−1^–1000 cm^−1^.

The metal–oxygen vibrational modes were examined using Fourier transform infrared (FTIR) transmission spectroscopy. Figure 3 represents the room-temperature FTIR spectra of the prepared LCMO samples measured over the wavenumber range of 390–1000 cm^−1^. From Figure 3, it can be observed that all the LCMO samples exhibited absorption peaks in the ranges of ~395–406 cm^−1^ and ~585–595 cm^−1^. The peaks in the ~395–406 cm^−1^ region correspond to the bending mode (υ_b_) of the Mn–O–Mn bond. The peaks in the ~585–595 cm^−1^ region correspond to the stretching mode (υ_s_) of the Mn–O or Mn–O–Mn bonds, thereby confirming the formation of metal–oxygen bonds [38].

From Figure 3, it is also evident that the stretching mode (υ_s_) peak broadened and showed a slight shift towards a higher wavenumber, with a decrease in intensity as the Ca^2+^ doping in the LCMO samples increased. The bending mode (υ_b_) peak showed a slight shift towards a lower wavenumber. Such changes are typically associated with distortions in bond lengths, bond angles or a combination of both [27,39]. The observed spectral changes are primarily attributed to reduced Jahn–Teller distortions with the increase in Ca^2+^ content, which subsequently altered the local Mn–O–Mn bond lengths [39,40].

**Figure 4:**
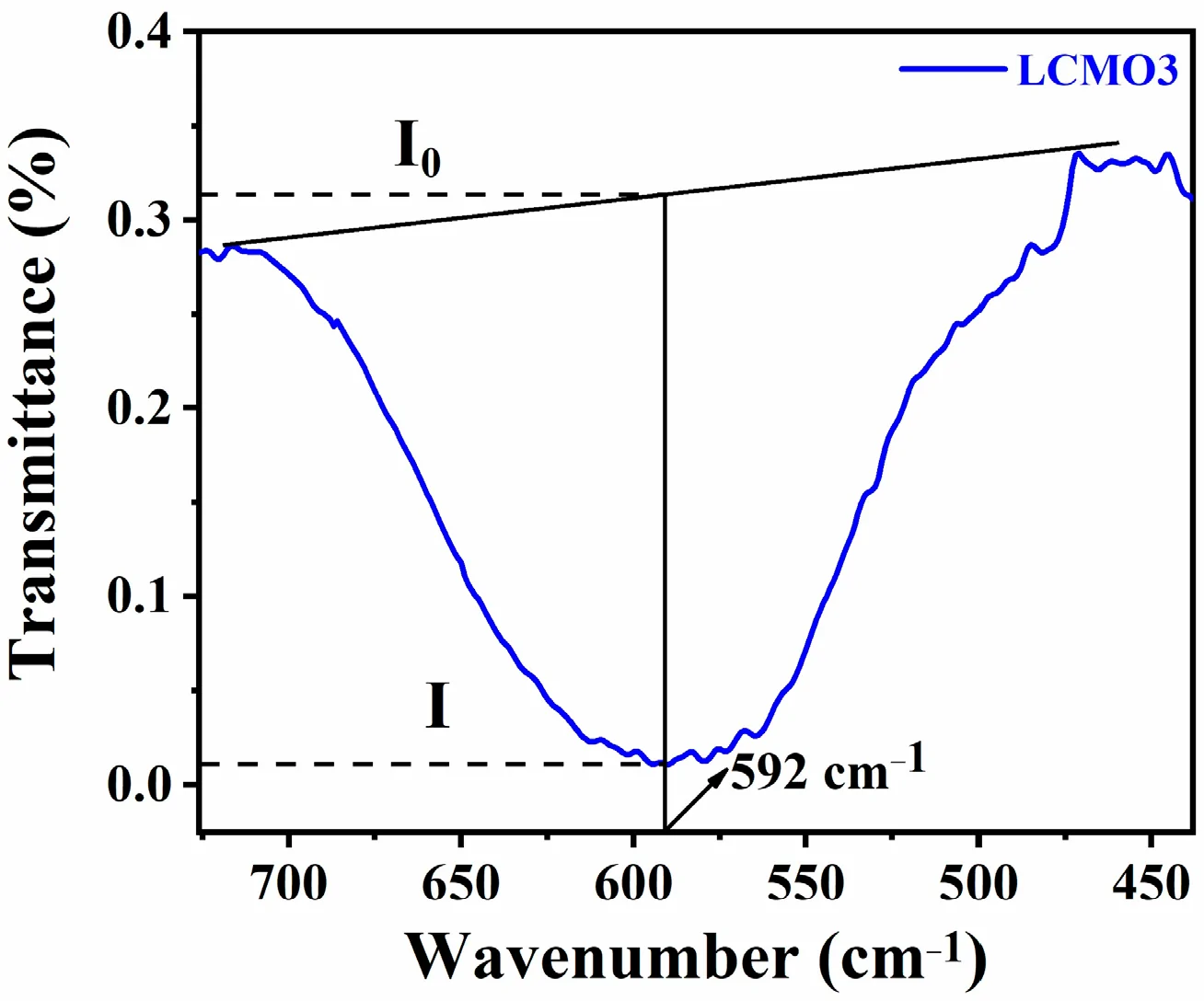
Graphic used to determine the absorbance values I and I_0_ for the FTIR peak of the LCMO3 sample.

In order to quantify the coupling of Mn–O–Mn bond and hence MnO_6_ octahedra, the Mn–O–Mn bond characteristics were systematically examined using the Beer–Bourger law, in accordance with established methodology reported in the literature [38,39]. This approach relates the absorbance ‘*A*’ of a peak to its transmittance ‘*T*’ through Equation (4):

(4) $$A=\log\left(\frac{1}{T}\right)=\log\left(\frac{I_{0}}{I}\right)$$

where I represents the maximum absorbance (minimum transmittance) of the vibrational peak, while I_0_ corresponds to the minimum absorbance (maximum transmittance) of the same peak. The calculation of I_0_ requires graphical procedure, as illustrated in Figure 4, and the resulting intensity ratios are provided in Table 3.

Table 3 indicates that the LCMO3 sample exhibited the highest absorbance, which gradually decreased with increasing Ca^2+^ content in the LCMO samples. This trend suggests a weakening of the Mn–O–Mn bond coupling as Ca^2+^ substitution increases. As the Ca^2+^ doping concentration in the LCMO samples increased, the vibrational coupling of the Mn–O–Mn bonds decreased, resulting in a reduction in IR absorption intensity [39]. This decrease in IR absorption intensity was accompanied by reduced MnO_6_ octahedral tilting with the increase in Ca^2+^ content, as observed in the XRD studies. The dependence of the FTIR absorption on Ca^2+^ doping makes these LCMO materials promising candidates for infrared detectors [41].

#### 2.1.3. Morphological Studies

**Figure 5:**
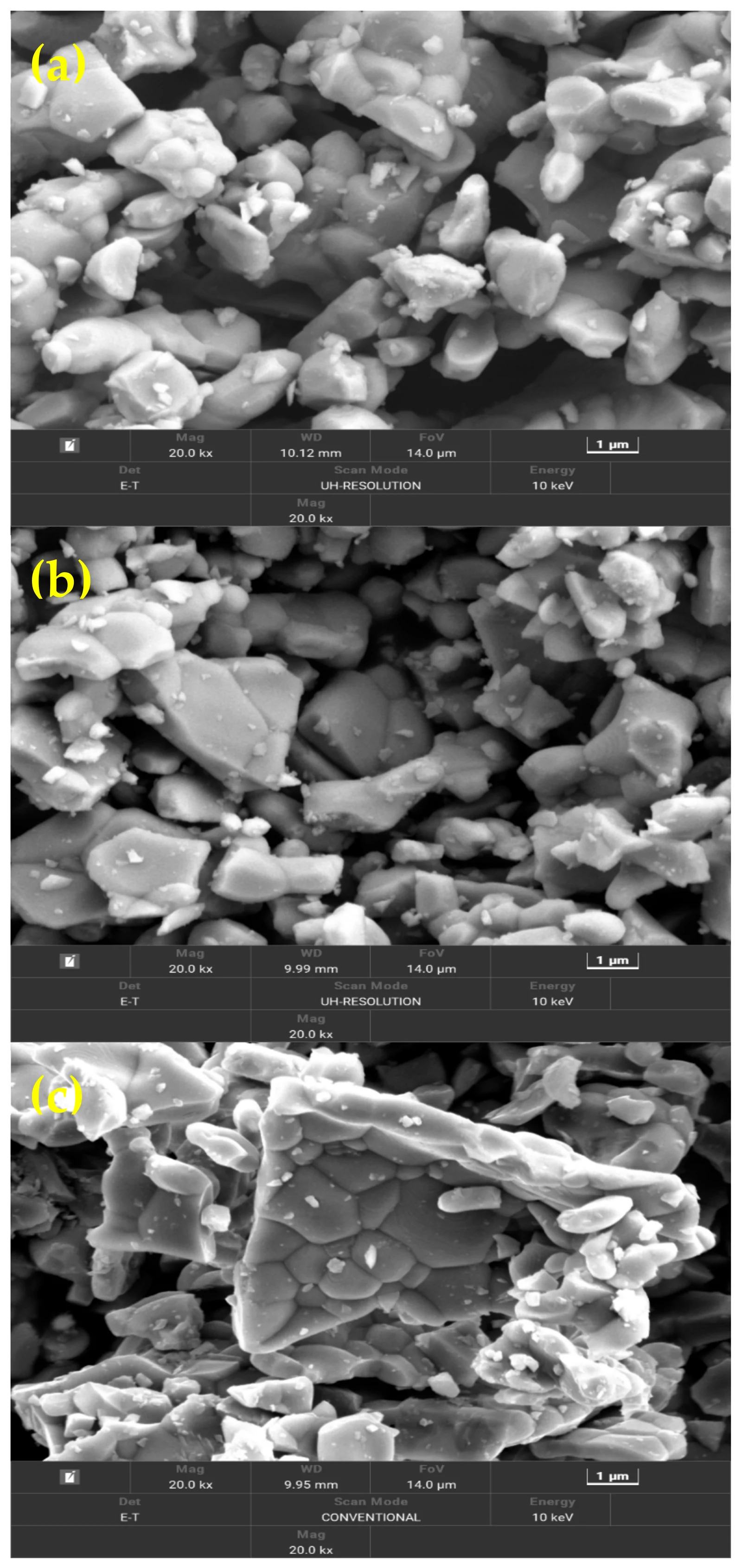
FESEM images of (**a**) LCMO3, (**b**) LCMO4 and (**c**) LCMO5 samples at 1 μm scale.

**Figure 6:**
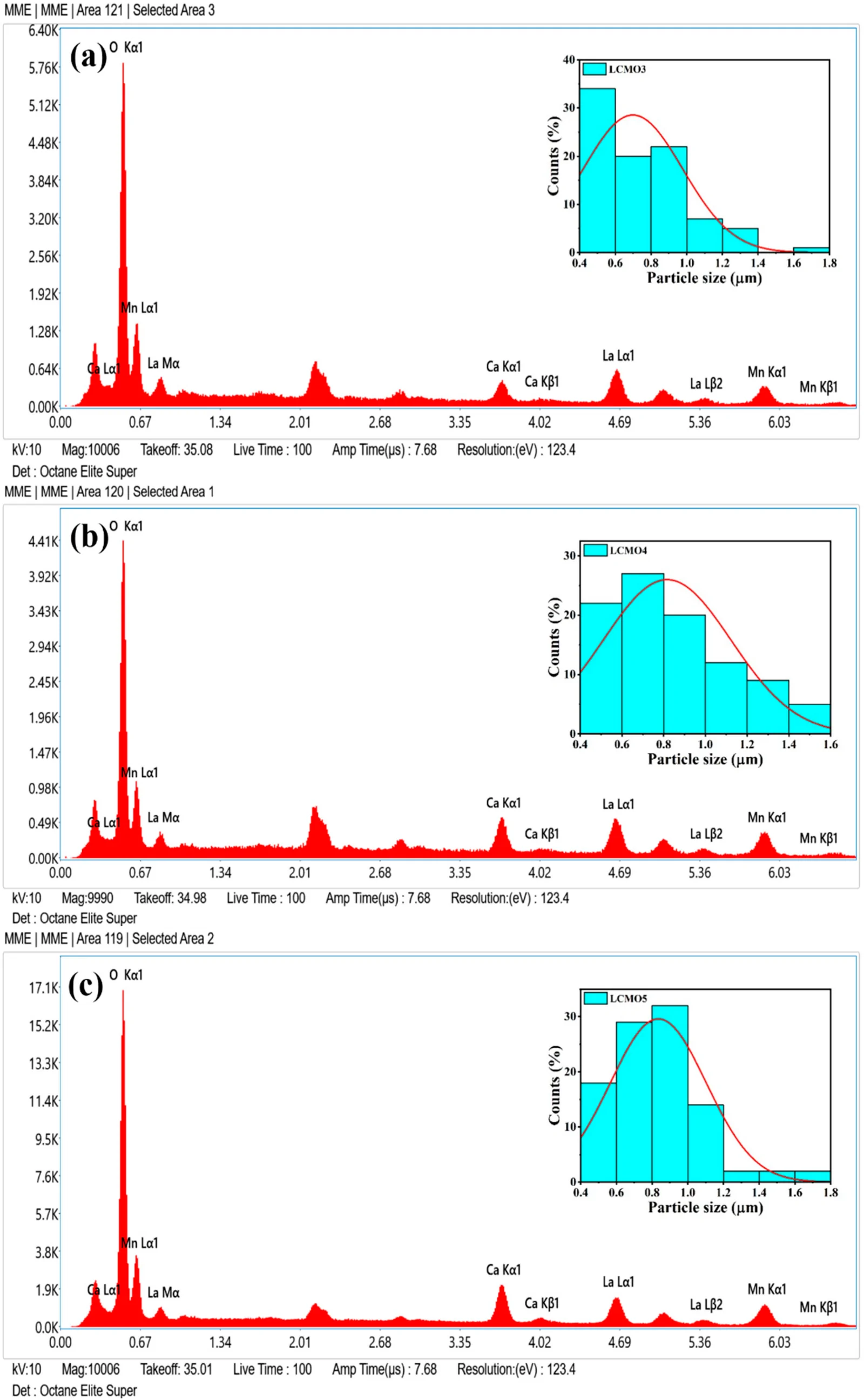
EDX spectra of the prepared (**a**) LCMO3, (**b**) LCMO4 and (**c**) LCMO5 samples showing their elemental composition, with insets depicting the corresponding particle size distributions. The red line in the figure indicates the normal distribution.

To investigate the surface morphology and elemental composition, the prepared LCMO samples were analysed using FESEM coupled with EDAX. Figure 5a–c represents the FESEM images of the LCMO3, LCMO4 and LCMO5 samples recorded at a 1 μm scale. The samples exhibit dense, irregularly shaped particles. Figure 6a–c shows the EDAX analysis of the LCMO samples, confirming La, Ca and Mn as the primary cationic constituents. The measured cationic ratios closely match the nominal compositions, indicating uniform incorporation of Ca^2+^ without the formation of secondary phases (Table 4). The insets of Figure 6a–c represent the corresponding particle size distribution plots for the LCMO3, LCMO4 and LCMO5 samples. The average particle sizes for LCMO3, LCMO4 and LCMO5 samples were 0.70 μm, 0.81 μm and 0.83 μm, respectively, indicating that the particle size increased with increasing Ca^2+^ doping. This trend is consistent with the crystallite size obtained from XRD studies, signifying that the synthesized samples were polycrystalline in nature [42]. The observed particle size obtained by FESEM was larger than the crystallite size obtained from XRD. This is because the particle size was acquired from a collection of multiple crystallites, whereas crystallite size corresponds to the coherently diffracting crystalline domain.

### 2.2. Magneto–Transport and Magnetoresistance (MR) Studies

#### 2.2.1. Magneto–Transport Studies

**Figure 7:**
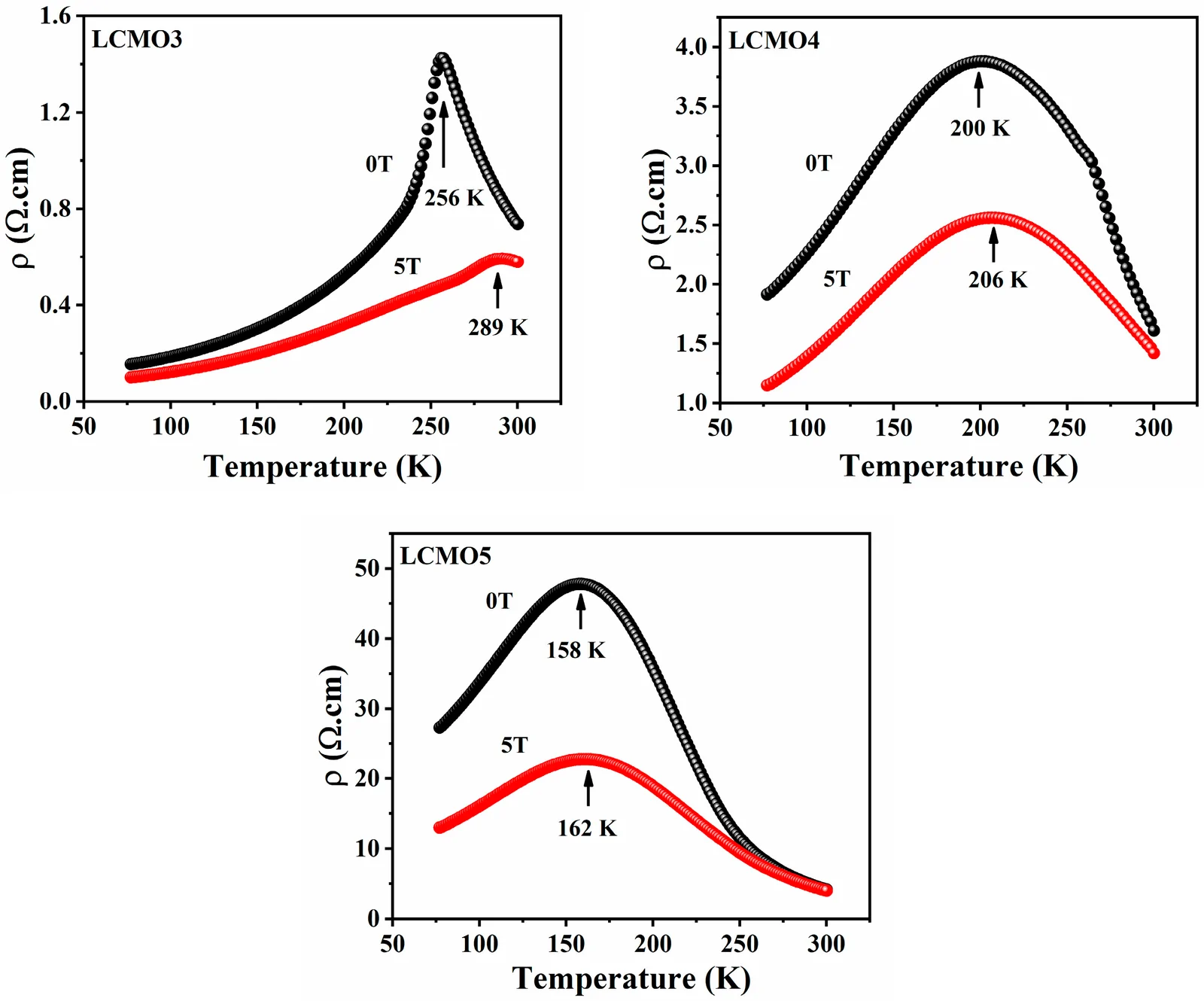
*ρ*–T plots of LCMO3, LCMO4 and LCMO5 samples measured from 77–300 K at both 0 T and 5 T magnetic fields.

Magneto–transport and MR studies were carried out to investigate the effects of temperature and external magnetic field on the charge transport properties. Temperature-dependent resistivity (*ρ*–*T*) measurements were recorded in the absence and presence of an applied magnetic field of 5 T in the temperature range 77–300 K. The *ρ* versus *T* plots at zero and 5 T fields are shown in Figure 7. From Figure 7, it can be observed that the resistivity of all the samples at all temperatures was smaller in the presence of the field compared to zero field. This is due to the fact that the applied magnetic field enhanced spin ordering, thereby reducing the resistivity [22]. Furthermore, in the low-temperature region, resistivity increased gradually with temperature, characteristic of a ferromagnetic nature. It reached its maximum at a particular temperature (T_MS_, metal–semiconductor/insulator transition temperature) and then decreased with the further increase in temperature, indicating a paramagnetic semiconducting/insulating nature [22].

From Figure 7, it can also be observed that the T_MS_ values for LCMO3, LCMO4 and LCMO5 at 0 T were 256 K, 200 K and 158 K, respectively, while at 5 T, they shifted to higher temperatures of 289 K, 206 K and 162 K. At 0 T and 5 T, the T_MS_ values were observed to decrease with the increase in Ca^2+^ concentration. This is due to the fact that as the Ca^2+^ content increased, the ferromagnetic state became less stable due to the increase in Mn^4+^ concentration and hence, the transition took place at a lower temperature [22]. The T_MS_ values for a given sample increased in the presence of magnetic field compared to zero field. This shift occurred because the applied magnetic field promoted local spin ordering and strengthened the double-exchange interaction. As a result, the ferromagnetic metallic state became more stable and dominated over the paramagnetic semiconducting/insulating state in the presence of the magnetic field [22].

To understand the variation of resistivity on either side of T_MS_, temperature–dependent resistivity models were employed.

In polycrystalline manganites, resistivity is primarily governed by two mechanisms at low temperature: (i) extrinsic spin-polarized tunneling (SPT) across grain boundaries and (ii) intrinsic double-exchange (DE) interaction within the grains [3]. Both extrinsic spin-polarized tunneling (SPT) and intrinsic double-exchange (DE) interactions coexist in the ferromagnetic metallic region and contribute to the resistivity. The role of SPT contribution to the resistivity in the low-temperature (ferromagnetic metallic) regions (77 < T < T_MS_) of the LCMO3, LCMO4 and LCMO5 samples was analysed using resistivity ρ(T,H) Equation (5) [17,18]:

(5) $$\rho (T,H)=\frac{r_{1}+r_{2}T^{\frac{3}{2}}}{1+\in <\cos\theta_{ij}>}$$

In the numerator of Equation (5), r_1_ and r_2_ are field independent parameters. In the denominator, ∈ is the spin polarization factor and cos θ_ij_ is the spin correlation function. The spin correlation function is given by Equations (6) and (7) in the absence and presence of applied magnetic field, respectively [17]:

(6) <cosθij>=−L|J|kTB

where L(x) = [coth(x) − 1/x] is the Langevin function and J is the antiferromagnetic exchange integral associated with the intergrain coupling.

(7) $$<\cos\theta_{ij}>=\frac{1}{4}+\frac{1}{3+\exp\left(\frac{-3J_{S}}{k_{B}T}\right)}$$

where J = S(S + 1). J_S_ represents the atomic ion spins.

**Figure 8:**
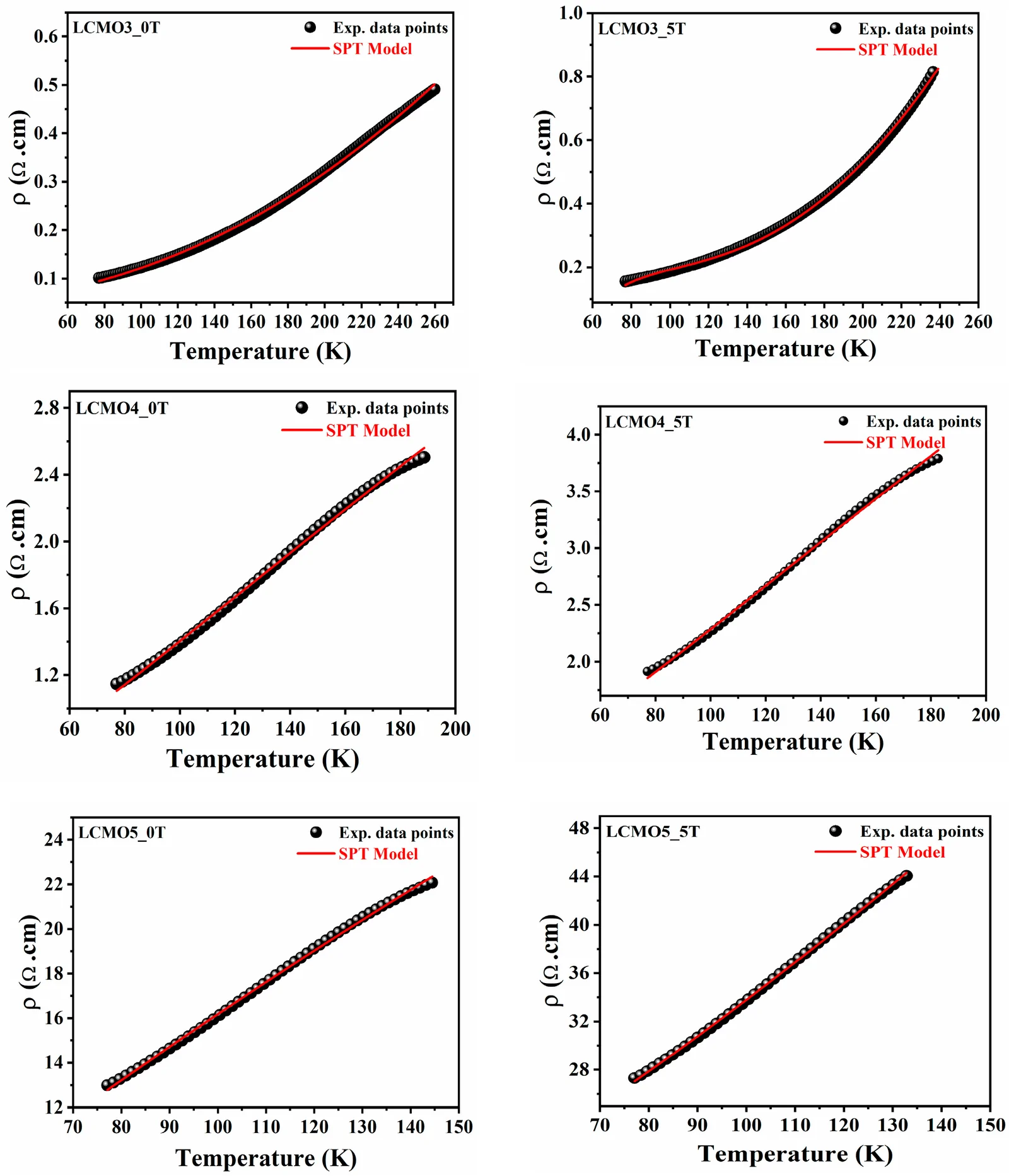
The low–temperature (ferromagnetic metallic) resistivity region of LCMO samples fitted using SPT model at 0 T and 5 T.

In the present work, the spin-polarized tunneling (SPT) model was first fitted to the experimental resistivity data measured at zero magnetic field (0 T) in the ferromagnetic metallic region below $T_{MS}$. Since no external magnetic field was present, the spin correlation function between neighboring grains was evaluated using the zero-field expression given by Equation (6) [17]. The fitted resistivity curves at 0 T are shown in Figure 8 and the field-independent parameters $r_{1}$ and $r_{2}$ were obtained from the fitting. The high regression coefficient (R^2^ = 0.99) for the fitted curves (Figure 8) indicates the reliability of fitted parameters.

The values of $r_{1}$ and $r_{2}$ obtained from the 0 T fitting were then used to calculate the resistivity at an applied magnetic field of 5 T by employing the field-dependent spin correlation function given in Equation (7). The fitted resistivity curves at 5 T are given in Figure 8. The good agreement (R^2^ = 0.99) between the calculated and experimental resistivity curves in the low-temperature region at 5 T confirms the dominance of intergrain spin-polarized mechanism. The applied magnetic field enhanced the spin alignment between neighboring grains, which increased the probability of tunneling across the grain boundaries and consequently reduced the resistivity in the ferromagnetic metallic region. However, the role of intrinsic double-exchange interaction contributing to the resistivity in this region cannot be neglected and was measured experimentally using ^55^Mn IFNMR at 77 K.

In the paramagnetic semiconducting/insulating region (T > T_MS_), the high-temperature resistivity of the prepared LCMO samples both at 0 T and 5 T was analyzed using the variable-range hopping (VRH) and small-polaron hopping (SPH) models [22]. The VRH model is applied for temperature ranges above T_MS_, and is studied in view of the localization of charge carriers and hopping processes [22]. The SPH model is applied for temperatures beyond the value of *θ_D_*/2, where *θ_D_* is the Debye temperature, and it considers the role of strong electron–phonon interactions and self-localization of charge carriers associated with the J–T distortions in the conduction mechanism [22]. In the present case, the temperature studies were carried out up to 300 K, where electron–phonon interactions are generally weak and hence, we ruled out the dominant role of SPH. The high-temperature resistivity of the prepared LCMO samples both at 0 T and 5 T was analyzed using the VRH model.

**Figure 9:**
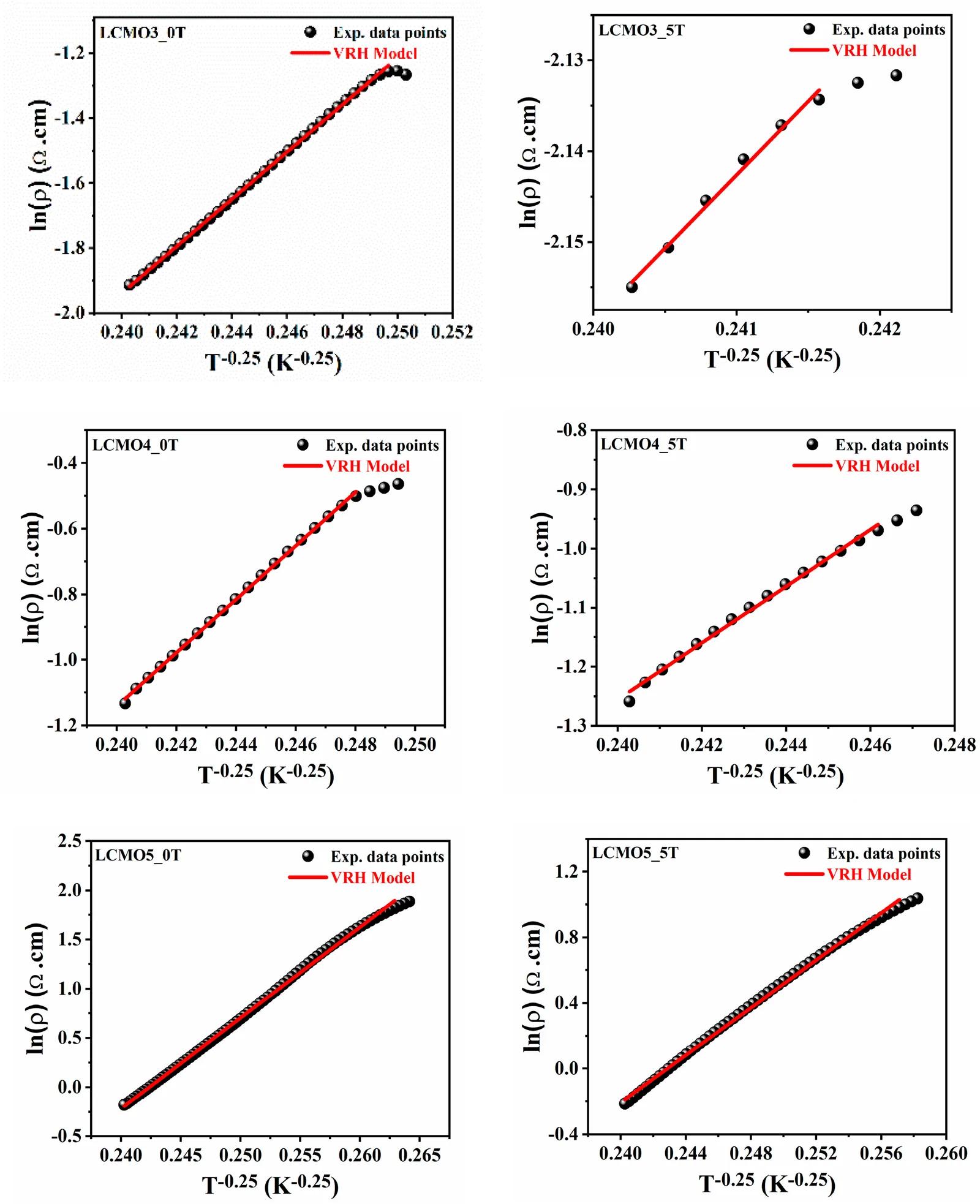
The high-temperature (paramagnetic semiconducting/insulating) resistivity region of the prepared LCMO samples fitted using the VRH model at 0 T and 5 T.

The VRH model utilizes Equation (8), which connects resistivity *ρ* at temperature *T* with Mott’s temperature (*T*_0_) and residual resistivity (*ρ_α_*) to describe the charge transport through hopping conduction mechanism. The fitted curves according to Equation (8) are shown in Figure 9. The high regression coefficient values (R^2^ = 0.99) obtained for all the LCMO samples confirm the reliability of the parameters obtained from the VRH model (Table 5). However, the errors which could have arisen from limited data points in the case of LCMO3 sample at 5 T should not be completely neglected.

The Mott temperature (*T*_0_), which is an inverse measure of probability of hopping obtained from the fitting is provided, in Table 5. The density of states at Fermi level *N (E_F_)*, estimated using Equation (9), is also given in Table 5.

(8) $$\rho =\rho_{\alpha }\exp{\left(\frac{T_{0}}{T}\right)}^{\frac{1}{4}}$$

(9) $$T_{0}=\frac{\lambda \alpha^{3}}{k_{B}N(E_{F})}$$

The mean hopping distance (*R_h_*) and hopping energy (*E_h_*) were also estimated using the Mott’s temperature (*T*_0_). The expressions for *R_h_* and *E_h_* are given in Equations (10) and (11), respectively [22,43,44]:

(10) $$R_{h}=\frac{3}{8}\alpha {\left(\frac{T_{0}}{T}\right)}^{\frac{1}{4}}$$

(11) $$E_{h}=\frac{1}{4}k_{B}T^{\frac{3}{4}}T_{0}^{\frac{1}{4}}$$

In the VRH model, *λ* = 18 is a dimensionless constant, *k_B_* = 8.617 × 10^−5^ eVK^−1^ is the Boltzmann constant, *T* is the absolute temperature, *h* is Planck’s constant and *α* = 2.50 nm^−1^ [44] is the inverse of localization length [44].

At 0 T, the characteristic temperature *T*_0_ increased from LCMO3 to LCMO5. This indicates the decrease in probability of hopping with the increase in Ca^2+^ content. This was due to a decrease in conducting *e_g_* electrons in Mn^3+^ ions with the increase in Ca^2+^ content. The decrease in density of states at the Fermi level *N (E_F_)* with the increase in Ca^2+^ content also supports the decrease in *e_g_* electrons. Furthermore, the mean hopping distance (*R_h_*) and hopping energy (*E_h_*) increased with the increase in Ca^2+^ content. This was due to breakdown of the DE interaction (Mn^3+^-O^2−^-Mn^4+^) with the increase in Ca^2+^ content. In the presence of an applied magnetic field of 5 T, the reduction in *T*_0_, *R_h_* and *E_h_* together with the increase in *N (E_F_)* indicates enhanced hopping probability due to improved spin alignment. Further, the applicability of the VRH model for the high temperature studied is confirmed by the product $R_{h}\alpha$. From Table 5, it can be observed that the product $R_{h}\alpha$ is greater than unity for all the LCMO samples, satisfying Mott’s criteria for the VRH conduction mechanism [43,44].

In summary, the magnetotransport results demonstrate that increasing Ca^2+^ content suppressed the metal–semiconductor transition temperature, whereas the application of a magnetic field shifted *T_MS_* to higher temperatures by promoting spin alignment. The conduction mechanism in the ferromagnetic metallic region (*T* < *T_MS_*) is satisfactorily described by the spin-polarized tunneling (SPT) model, indicating the role of spin-dependent intergrain tunneling. Above *T_MS_*, the conduction mechanism is due to the hopping of charge carriers from one localized site to another, governed by the VRH model.

#### 2.2.2. Magnetoresistance (MR) Studies

**Figure 10:**
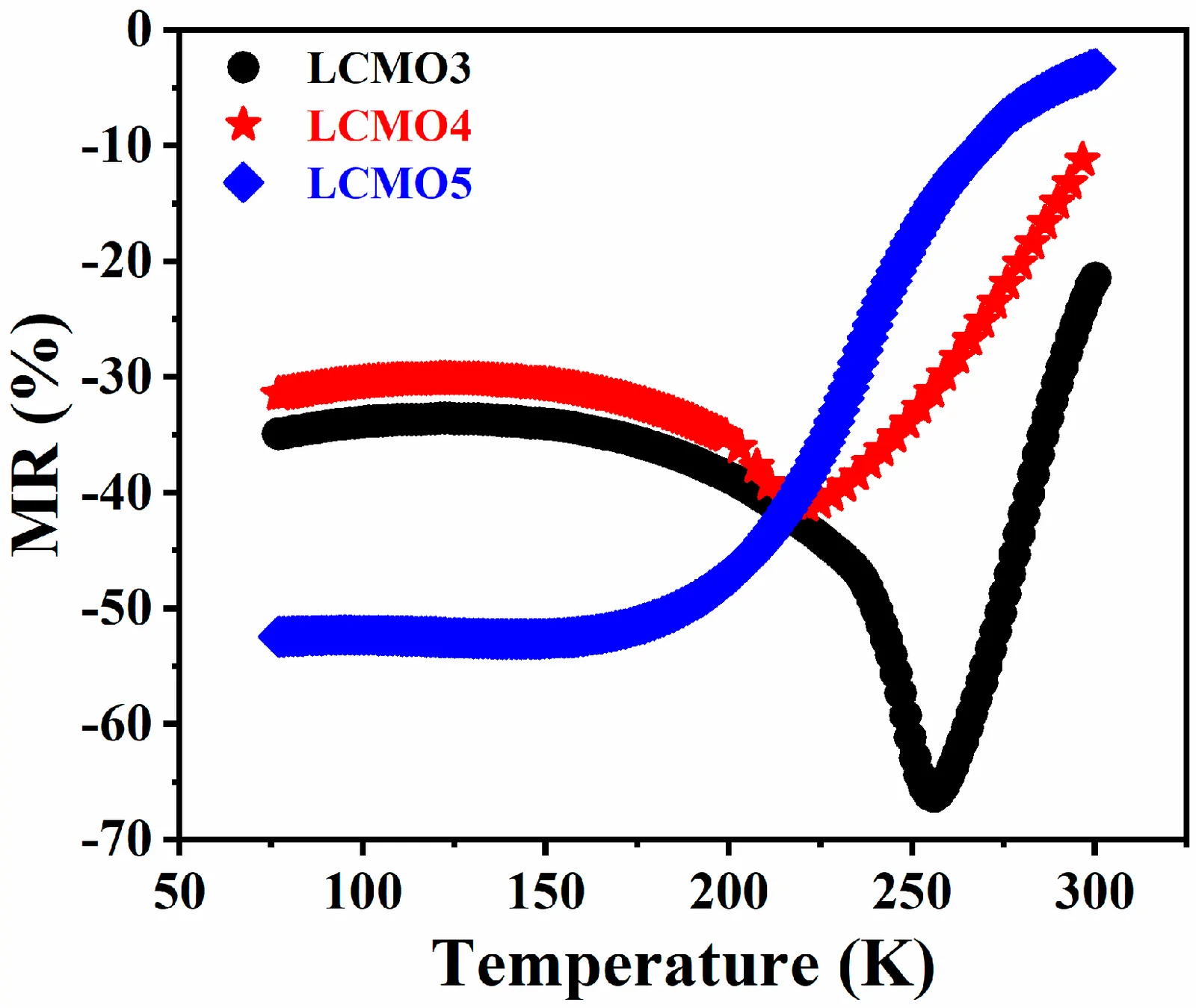
Temperature dependent of magnetoresistance (MR%) of LCMO samples.

The noticeable reduction in resistivity in the presence of applied magnetic field of 5 T of LCMO samples, observed in both the low-temperature (T < T_MS_) and high-temperature (T > T_MS_) regions (Section 2.2.1), gave rise to a magnetoresistance (MR) effect. Figure 10 shows the variation of MR (%) for LCMO samples in the presence of 5 T magnetic field in the temperature range of 77–300 K. The MR (%) of LCMO samples was calculated using Equation (12) [45]. In this equation, *ρ*(5) and *ρ*(0) represents the resistivity measured at magnetic fields of 5 T and 0 T, respectively.

(12) $$MR(\%)=\frac{\rho (5)-\rho (0)}{\rho (0)}\times 100$$

Figure 10 shows that the maximum MR was observed near *T_MS_* for LCMO3 and LCMO4, whereas LCMO5 did not exhibit a distinct MR peak. In polycrystalline manganites, the observed magnetoresistance (MR) is primarily governed by two mechanisms at low temperature: (i) extrinsic spin-polarized tunneling (SPT) across grain boundaries and (ii) intrinsic double-exchange (DE) interaction within the grains [3]. Both extrinsic spin-polarized tunneling (SPT) and intrinsic double-exchange (DE) interactions coexist in the ferromagnetic metallic region and contribute to the magnetoresistance (MR) [3].

At 77 K, the MR values were 34.91%, 31.49% and 52.64% for LCMO3, LCMO4 and LCMO5, respectively. Since the samples are in the ferromagnetic metallic region, the low-temperature MR was governed by spin-polarized tunneling (SPT) across grain boundaries, which was observed in the magnetotransport studies. The applied magnetic field enhanced spin alignment between neighbouring grains, increased the probability of tunneling and consequently reduced the grain–boundary resistance.

Near *T_MS_*, the MR increased to 66.26% for LCMO3 and 41.09% for LCMO4, due to the suppression of spin disorder by the applied magnetic field. Around the magnetic transition, the field strengthened the double-exchange (DE) interaction by improving the alignment of neighbouring Mn spins, thereby enhancing carrier mobility and producing the maximum MR [3]. In contrast, LCMO5 did not show a strong MR peak near *T_MS_* (162 K) and its MR remained nearly the same as at 77 K (52.64%), suggesting the presence of magnetic inhomogeneities and competing interactions [46,47,48]. This behavior is often linked to the Griffiths phase in half–doped manganites (LCMO5), where ferromagnetic clusters exist within a paramagnetic matrix due to stronger spin disorder caused by ionic mismatch at the A site, disrupting long-range order [48]. Additionally, periodic charge ordering of Mn^3+^ and Mn^4+^ ions in LCMO5 formed stable conduction paths, resulting in nearly constant MR across the ferromagnetic region [49]. This is in consistent with reported studies [48,49,50].

Above *T_MS_*, the MR decreased to 21.47%, 11.23% and 3.37% at 300 K for LCMO3, LCMO4 and LCMO5, respectively. The observed magnetoresistance originated primarily from the magnetic field induced enhancement of carrier hopping rather than long-range ferromagnetic ordering. At high temperatures (above *T_MS_*), the MR is governed by hopping of charge carriers from one localized site to another, in accordance with the VRH model [22].

### 2.3. Mn Oxidation State and Magnetization Studies

The magneto transport and MR studies of the LCMO samples indicated the role of the double-exchange (DE) mechanism below and around T_MS_. Furthermore, these studies also revealed the paramagnetic nature of the LCMO samples above T_MS_. The DE mechanism originates from the hopping of *e_g_* electrons between neighbouring Mn^3+^ and Mn^4+^ ions through the Mn^3+^–O^2−^–Mn^4+^ interaction, which simultaneously couples electronic transport with ferromagnetic spin alignment [12] and strongly depends on the distribution of Mn oxidation states. The distribution of Mn oxidation states was examined using X-ray photo electron spectroscopy (XPS) at 300 K and ^55^Mn internal field nuclear magnetic resonance (IFNMR) at 77 K. The VSM studies at 300 K (above T_MS_) were carried out to examine the paramagnetic behavior and its variation with increasing Ca^2+^ content.

#### 2.3.1. X-Ray Photo Electron Spectroscopy (XPS) Studies

The XPS studies were carried out for LCMO3, LCMO4 and LCMO5 samples to examine their chemical composition and the distribution of Mn oxidation states (Mn^3+^ and Mn^4+^). The survey spectra (Figure S2) (Supplementary Materials) show the La 3d, Ca 2p, Mn 2p and O 1s core levels, confirming the presence of all the elements in LCMO samples. The observed peak at 285 eV is attributed to carbon 1s and was used as an internal reference for binding energy calibration [26,27]. No distinct spectral features corresponding to Mn^2+^ species were observed near ~639 eV, suggesting the absence of significant Mn^2+^ contribution in the prepared samples [27].

The Mn 2p core–level spectra were further deconvoluted to identify Mn^3+^ and Mn^4+^ contributions. Peak fitting was carried out using XPS Peak Fit 4.1 with Gaussian–Lorentzian line shapes and a Shirley background (Figure S3a–c) (Supplementary Materials) [26,27]. The fitted peak positions and corresponding areas are listed in Table 6. The Mn 2p_3/2_–Mn 2p_1/2_ energy separation values (11.64, 11.57 and 11.32 eV for LCMO3, LCMO4 and LCMO5 respectively) confirm the coexistence of Mn valence states (Mn^3+^ and Mn^4+^) [26,27].

Interpreting the O 1s spectra of manganites is often difficult due to conflicting reports in the literature. While some studies describe the spectrum as consisting of three components, others argue that only two should be considered [51,52]. To address these inconsistencies in distinguishing the oxygen bonding environment, the O 1s spectra of the synthesized LCMO samples were deconvoluted. The resulting spectra are presented in Figure S3d–f and the corresponding fitting parameters are listed in Table 7.

The cumulative fitted curves in Figure S3a–c align closely with the experimental Mn 2p core–level spectra. Similarly, in Figure S3d–f, the cumulative fit shows excellent agreement with the experimental O 1s spectra, further demonstrating the quality and accuracy of the extracted fitting parameters (Table 6 and Table 7).

Table 6 shows that, in all the Ca^2+^-doped LCMO samples, the Mn^3+^ peaks appeared at lower binding energies, while the Mn^4+^ peaks shifted to higher binding energies. Table 7 reveals that with increasing Ca^2+^ doping in LCMO samples, the Mn^4+^ content increased while Mn^3+^ decreased, resulting in a higher Mn^4+^/Mn^3+^ ratio. The experimental Mn^4+^/Mn^3+^ ratios for the LCMO samples were lower than the nominal values, indicating the presence of oxygen vacancies [52]. The oxygen vacancy concentration in LCMO3 was estimated to be 18.60%, which decreased to 15.15% in LCMO4 and 6.00% in LCMO5 with the increasing Ca^2+^ content.

Table 7 shows that the Mn–O contribution appeared near ~529 eV (O1), while that of La–O was observed around ~531 eV (O2), consistent with previous reports [47,53]. The decrease in the O2 component with increasing Ca^2+^ content in the LCMO samples indicates that substitution of Ca^2+^ reduced the number of La^3+^ sites, thereby diminishing the La–O contribution.

#### 2.3.2. ^55^Mn Internal Field Nuclear Magnetic Resonance (IFNMR) Studies

**Figure 11:**
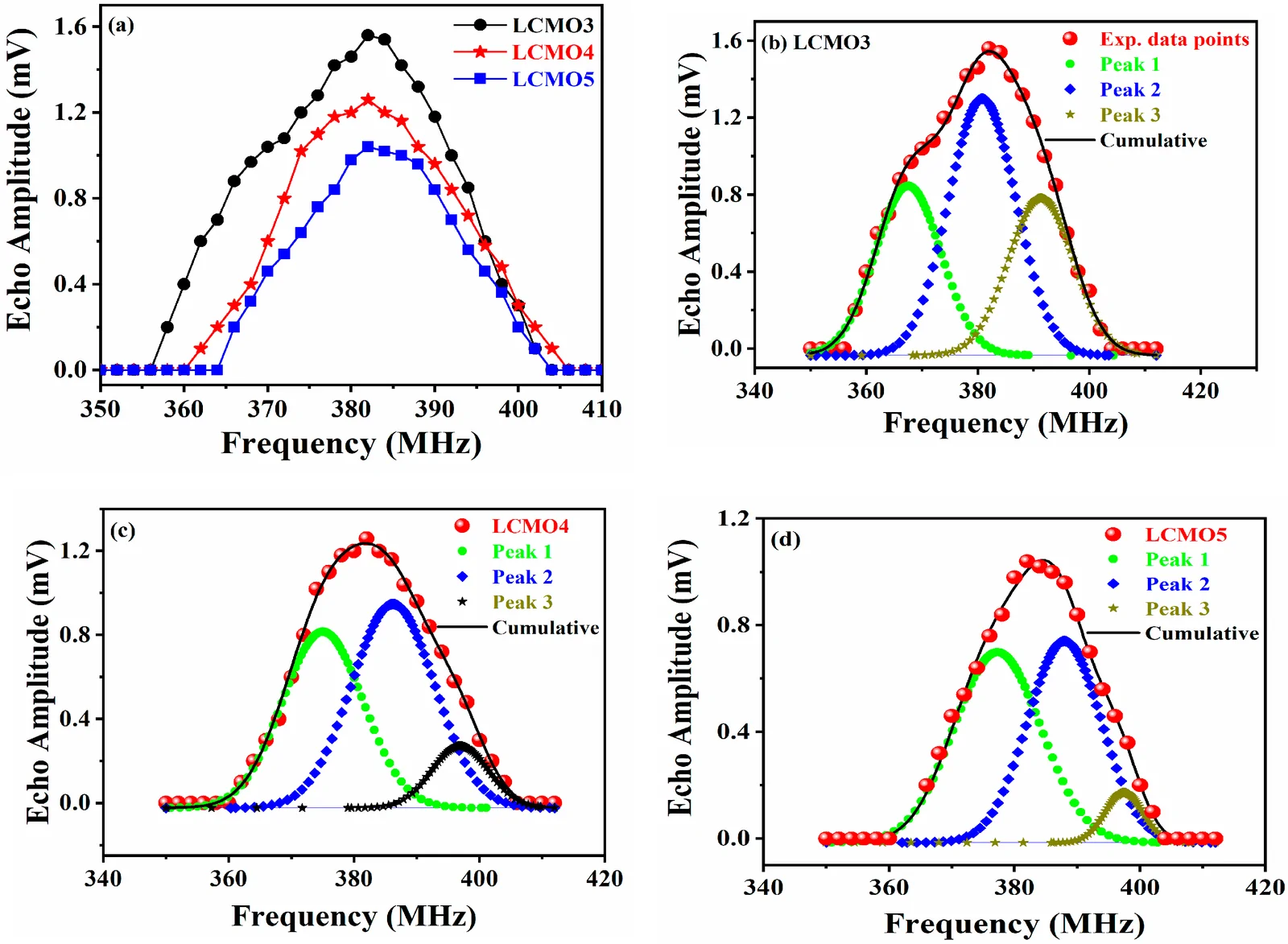
(**a**) ^55^Mn IFNMR spectra of all the prepared LCMO samples and (**b**–**d**) are the corresponding deconvoluted spectra of LCMO3, LCMO4 and LCMO5.

The ^55^Mn IFNMR studies were performed at 77 K, to investigate the role of intrinsic double-exchange (DE) interaction in relation to the resistivity and its correlation with the magnetoresistance (MR) behavior as a function of Ca^2+^ substitution in LCMO. As a local probe, ^55^Mn IFNMR directly senses the hyperfine magnetic field, providing detailed information on the local magnetic environment and the strength of the DE interaction. Furthermore, the technique effectively distinguishes the Mn^3+^ and Mn^4+^ oxidation states, enabling a comprehensive understanding of their roles in governing the magnetic and magnetotransport properties of LCMO [26,27]. The ^55^Mn IFNMR spectra of the prepared LCMO samples at 77 K are shown in Figure 11a. This figure reveals broad, asymmetric resonance lines for all LCMO samples, confirming ferromagnetic metallic nature at 77 K. With the increase of Ca^2+^ content in LCMO samples, the IFNMR spectrum becomes broader and the echo amplitude progressively decreases.

The internal or hyperfine magnetic field (H) in magnetic materials originates from multiple contributions, as mentioned in the reported literature [32,54]. The internal magnetic field at the ^55^Mn nucleus originates from hyperfine interactions, which primarily include the Fermi contact interaction, transferred hyperfine interaction, dipolar interaction and orbital contribution. Among these, the Fermi contact interaction is the dominant contribution, while the transferred hyperfine interaction reflects the magnetic exchange between neighboring Mn ions through the Mn^3+^-Mn^4+^ network [54]. The resonant IFNMR frequency for ^55^Mn nuclei can be determined using Equation (13) [27,33,36]:

(13) $$f=\frac{\gamma H}{2\pi }$$

where *γ* represents the gyromagnetic ratio (10.553 MHzT^−1^ for ^55^Mn). Using this relation, the estimated resonance frequency lies in the range of 340–410 MHz.

The broad asymmetric spectrum shown in Figure 11a was deconvoluted into three Gaussian peaks (*f*_1_, *f*_2_ and *f*_3_), reflecting the inhomogeneity in the local environment of Mn ions participating in high-frequency Mn^3+^/Mn^4+^ electron exchange [55,56,57]. The deconvoluted spectra of all the prepared LCMO samples, each separated into three components, are presented in Figure 11b–d. The corresponding fitted parameters are provided in Table 8.

In this study, the three deconvoluted frequency components were assigned based on earlier reports [27]. The low-frequency peak at *f*1 = 367–377 MHz was assigned to Mn environments with an effective average valence close to (Mn^~3.8+^) and a magnetic moment of 3.00 μB, which are likely to be surrounded by Ca^2+^ ions and cationic vacancies to maintain local charge neutrality [56]. The dominant central peak at *f*2 = 380–388 MHz is attributed to fast electron (or hole) hopping between Mn sites with an effective average valence of approximately μ (Mn^~3.5+^) = 4.38 μB in ferromagnetic metallic (FMM) regions. This electron hopping occurs at a rate exceeding the NMR (Larmor) frequency due to the double-exchange (DE) interaction [57]. The high-frequency component at *f*3 = 391–397 MHz, arises from partially localized Mn states with an effective average valence close to (Mn^~3.17+^) and a magnetic moment of μ = 4.73 μB, where anion vacancies disrupt the DE interaction and reduce the effective manganese valence [24].

From Table 8, it is evident that the area corresponding to *f*1 increased with Ca^2+^ doping, indicating a larger proportion of high-valence Mn^4+^ ions. In contrast, the area of *f*2 remained almost unchanged with increasing Ca^2+^ doping, suggesting that the double-exchange (DE) interaction in the LCMO samples was largely preserved. The decreasing area of *f*3 is attributed to the reduction of partially localized Mn^3+^ states associated with nearby anion vacancies. This trend is in good agreement with the reduction of oxygen vacancies estimated from XPS studies.

Table 9 shows that the Mn^4+^/Mn^3+^ valence ratios obtained from XPS and ^55^Mn IFNMR are in excellent agreement with the nominal compositions for La_1−*x*_Ca*_x_*MnO_3_ samples, confirming the consistency between bulk-sensitive IFNMR and surface-sensitive XPS measurements. Although the Mn^4+^/Mn^3+^ ratio increased systematically with Ca^2+^ content, the IFNMR results reveal the DE strength, which is evident from the area of the central *f*2 (ferromagnetic metallic, FMM) component. This indicates that the fundamental Mn^3+^–O^2−^–Mn^4+^ hopping network responsible for DE interaction was preserved even at higher Ca^2+^ doping levels, maintaining the integrity of the ferromagnetic metallic phase. Importantly, the partial localization component *f*3, associated with ~Mn^3+^ states influenced by anion vacancies, decreased markedly with increasing Ca^2+^ doping.

**Figure 12:**
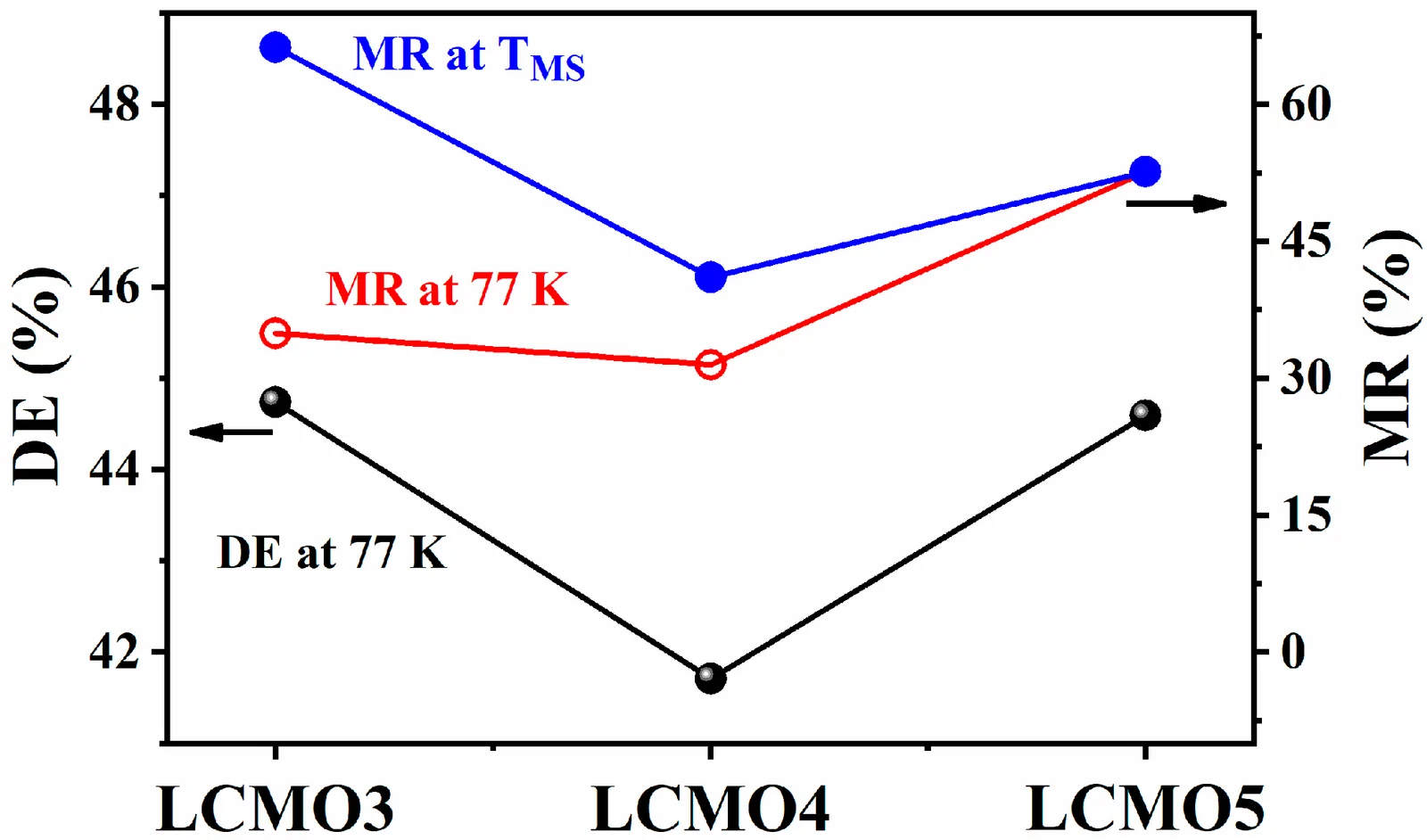
Variation of double-exchange (DE) strength with MR%at 77 K and at *T_MS_* for LCMO samples.

The variation of MR in both regions shows good agreement with the DE strength obtained from ^55^Mn IFNMR at 77 K (Figure 12), confirming the strong correlation between the ferromagnetic metallic DE network and the magnetotransport behavior. Thus, the ^55^Mn IFNMR technique, which probes the strength of the DE interaction, serves as a valuable indirect tool for investigating MR through the detection of DE behaviour. Furthermore, the correlation between the DE strength obtained from ^55^Mn IFNMR and the observed MR is consistent with our earlier reports [26,27].

#### 2.3.3. Vibrating Sample Magnetometry (VSM) Studies

**Figure 13:**
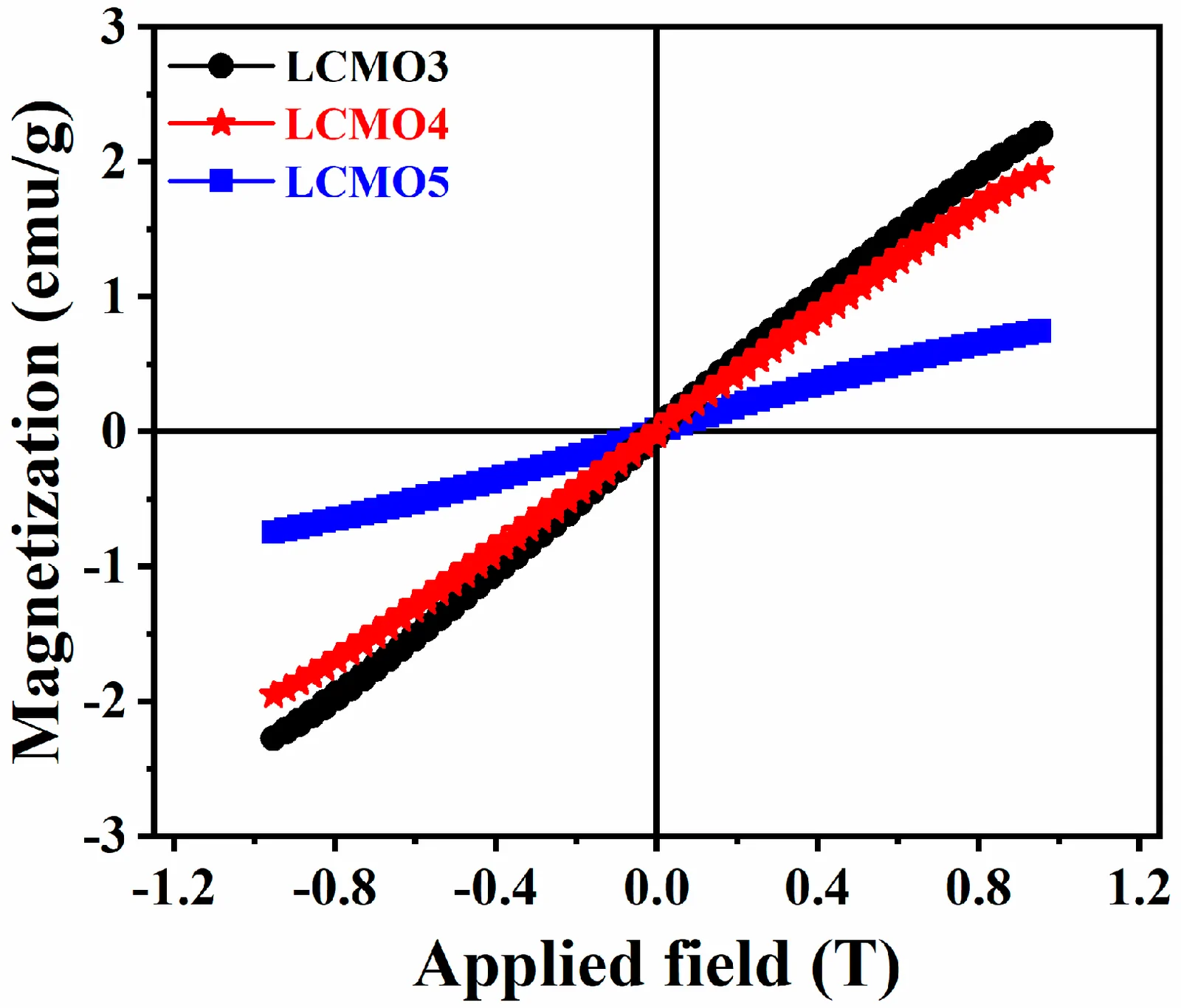
Room temperature (M–H) curves of LCMO samples in the range of −1 T to 1 T.

Figure 13 shows the magnetization (M–H) curves of the LCMO3, LCMO4 and LCMO5 samples. The curves exhibit a linear dependence on the applied field, with no remanence or coercivity, confirming that all the samples were paramagnetic at room temperature. The magnetic susceptibilities (*χ*) measured at 1 T were 2.20 emu g^−1^ T^−1^ for LCMO3, 2.00 emu g^−1^ T^−1^ for LCMO4 and 0.75 emu g^−1^ T^−1^ for LCMO5.

LCMO3 (*x* = 0.3) showed the highest susceptibility, and a clear decline in *χ* was observed with increasing Ca^2+^ doping. However, as the Ca^2+^ doping increased further (in samples *x* = 0.4 and 0.5), susceptibility χ decreased. The decrease in *χ* further supports the decrease in transition temperature with increasing Ca^2+^ content that was evident from the MR studies [58]. This supports the assertion that the ferromagnetic metallic state is progressively destabilized as the double-exchange mechanism breaks, a trend that is consistent with observations reported in the literature [58,59].

## 3. Materials and Methods

### 3.1. Materials

La(NO_3_)_3_·6H_2_O (extra pure, AR 99.9%, Loba Chemie Pvt. Ltd., Mumbai, India), Ca(CH_3_COO)_2_·xH_2_O (AR 99%, SRL Pvt. Ltd., Shivmogga, India) and Mn(CH_3_COO)_2_·4H_2_O (extra pure, AR 99%, SRL Pvt. Ltd., Shivmogga, India) were used as starting materials (precursors). Ethylene glycol (extra pure, AR 99%, SRL Pvt. Ltd., Shivmogga, India) was used as the surfactant, while glycine (extra pure, AR 98.5%, Loba Chemie Pvt. Ltd., Bangalore, India) was used as a fuel. Deionized water (Medilise Chemicals, Ballari, India) was used to dissolve the precursors and liquor ammonia (Qualigens, Ballari, India) was added to control the pH.

### 3.2. Methods

#### 3.2.1. Synthesis

La_1−*x*_Ca*_x_*MnO_3_ (*x* = 0.3, 0.4 and 0.5) samples were synthesized via a sol–gel method. The precursors mentioned in the Section 3.1 were dissolved in deionized water while maintaining a metal cation–ethylene glycol–glycine molar ratio of 1:2:2 to promote chelation. The pH of the solution was maintained at 7.0 by the gradual addition of liquor ammonia. The mixture was then heated on a magnetic stirrer at 80 °C with 300 rpm until a gel was formed. The resulting gel was combusted at 180 °C for 2 h, yielding a fluffy reddish–brown powder. This powder was subsequently calcined at 1000 °C for 5 h to obtain the final dark brown product. The synthesized samples with Ca^2+^ concentrations of *x* = 0.3, 0.4 and 0.5 are referred to as LCMO3, LCMO4 and LCMO5, respectively.

#### 3.2.2. XRD, FTIR and FESEM

The phase purity and crystal structure of La_1−*x*_Ca*_x_*MnO_3_ samples were examined using X-ray diffraction (XRD) (Bruker D2 Phaser, Cu–Kα, λ = 0.154 nm). Rietveld refinements were performed using the *Full Prof* program [60]. Crystallite size and microstrain were estimated using the Halder–Wagner–Langford method [61]. FTIR spectra were recorded on a PERKIN ELMER Spectrum 2 spectrometer (PerkinElmer, Shelton, CT, USA) using 12 mm KBr pellets. Microstructural and compositional analyses were carried out using a field emission scanning electron microscope (FESEM) (CLARA model, TESCAN, Brno, Czech Republic) equipped with an energy-dispersive X–ray analysis (EDAX) OCTANE ELITE detector (AMETEK Inc., Pleasanton, CA, USA).

#### 3.2.3. MR, VSM, XPS and ^55^Mn IFNMR

Magnetoresistance (MR) studies were carried out through resistivity measurements both in the absence and presence of applied magnetic field (5 T), using a PPMS Dynacool D212 system (Quantum Design, San Diego, CA, USA) with silver contacts on pelletized samples (7 mm × 2 mm × 2 mm) over a temperature range of 77–300 K. Room temperature magnetic measurements (M–H curves) were measured with a home-built vibrating sample magnetometer (VSM) developed by Pratheek et al. [62]. X-ray photoelectron spectroscopy (XPS) survey scans were obtained with a Thermo Fisher Scientific system (Al–Kα source) (Waltham, MA, USA) and Mn 2p spectra were fitted using XPSPEAK 4.1 with a Shirley background [55] to quantify the Mn^4+^/Mn^3+^ ratio. ^55^Mn internal field nuclear magnetic resonance (IFNMR) measurements were conducted at 77 K using a home-built pulsed spectrometer [63], employing a two equal pulse spin–echo sequence (π/2–τ–π/2) to detect weak and short duration signals [64,65,66,67]. Spin–echo amplitudes were recorded as a function of frequency over 340–410 MHz with an interval of 2 MHz. A hand-held Rig Expert (AA–600 model) impedance analyser (Rig Expert Ukraine Ltd., Kyiv, Ukraine) was used to tune the signal at each frequency.

## Figures and Tables

**Table 1:** Structural parameters of LCMO samples obtained from Rietveld refinement.

Parameters	Samples		
	LCMO3	LCMO4	LCMO5
*a* (Å)	5.47586 (7)	5.44270 (1)	5.43623 (4)
*b* (Å)	7.77509 (1)	7.72413 (6)	7.66403 (1)
*c* (Å)	5.50201 (2)	5.46146 (8)	5.43412 (5)
*V* (Å^3^)	234.249 (1)	229.600 (4)	226.404 (6)
*α = β* = *γ* (^0^)	90	90	90
*a_p,the_* (Å)	4.021	3.998	3.975
*a_p,ref_* (Å)	3.083	3.062	3.048
Δ*a_p_* (Å)	0.938	0.936	0.927
*R_wp_ (%)*	5.87	5.69	5.51
*R_p_ (%)*	4.67	4.33	4.14
*R_exp_ (%)*	4.85	4.27	3.95
*R_Bragg_ (%)*	4.65	4.64	4.64
*χ* ^2^	1.465	1.781	1.945

**Table 2:** Microstructural parameters estimated from the HWL plot, along with the dislocation density.

Samples	*D* (nm)	*ε* × 10^−3^	*δ* × 10^−3^ (nm^−2^)
LCMO3	11.13	9.76	8.07
LCMO4	12.95	7.77	5.96
LCMO5	13.36	6.24	5.60

**Table 3:** The intensity ratios calculated using the Beer–Bourger law.

Samples	*I*	*I*_0_	*I*_0_ */I*	A
LCMO3	0.01	0.31	31.00	1.49
LCMO4	2.12	4.73	2.23	0.35
LCMO5	0.37	0.70	1.89	0.27

**Table 4:** The nominal and measured elemental compositions of the LCMO samples.

Sample		La	Ca	Mn	Error (%)
LCMO3	Nominal	0.70	0.30	1.00	1.92
	Elemental	0.69	0.31	0.99	
LCMO4	Nominal	0.60	0.40	1.00	4.11
	Elemental	0.58	0.42	1.04	
LCMO5	Nominal	0.50	0.50	1.00	4.66
	Elemental	0.51	0.49	1.10	

**Table 5:** Parameters obtained from paramagnetic semiconducting/insulating region (T > T_MS_) of LCMO3, LCMO4 and LCMO5 samples using the VRH model at 0 T and 5 T.

Parameters	LCMO3		LCMO4		LCMO5	
	0 T	5 T	0 T	5 T	0 T	5 T
*R* ^2^	0.99	0.99	0.99	0.99	0.99	0.99
*T*_0_ (10^7^ K)	2.76	0.02	4.45	0.53	7.21	2.73
*N (E_F_)* (10^23^ eV^−1^ cm^3^)	1.68	17.48	1.04	8.75	0.64	1.70
*R_h_* (Å)	6.57	4.52	6.77	5.92	6.98	6.56
*E_h_* (meV)	113.79	38.14	128.23	103.05	144.71	120.25
*R_h_* *α*	1.64	1.13	1.69	1.32	1.74	1.64

**Table 6:** Mn 2p peak positions, corresponding peak areas and the experimental and nominal Mn^4+^/Mn^3+^ valence ratios for all the LCMO samples.

Sample	Spin–Orbit Split	Peak Position (eV)	Mn States	Peak Position (eV)	Area (%)	Mn^4+^/Mn^3+^ Ratio	
						Exp.	Nominal
LCMO3	Mn 2p_3/2_	641.78	Mn^3+^	641.05	49.13	0.35	0.43
			Mn^4+^	642.58	17.54		
	Mn 2p_1/2_	653.42	Mn^3+^	652.00	24.56		
			Mn^4+^	653.88	8.77		
LCMO4	Mn 2p_3/2_	641.90	Mn^3+^	641.11	42.59	0.56	0.66
			Mn^4+^	642.63	24.07		
	Mn 2p_1/2_	653.47	Mn^3+^	652.75	21.30		
			Mn^4+^	654.19	12.04		
LCMO5	Mn 2p_3/2_	642.03	Mn^3+^	640.99	32.87	0.94	1.00
			Mn^4+^	642.48	34.21		
	Mn 2p_1/2_	653.35	Mn^3+^	652.65	16.24		
			Mn^4+^	654.04	17.09		

**Table 7:** Peak positions and area percentages derived from the deconvolution of O 1s curve.

Peaks	LCMO3		LCMO4		LCMO5	
	Peak Position (eV)	Area (%)	Peak Position (eV)	Area (%)	Peak Position (eV)	Area (%)
O1	529.16	34.33	529.06	35.70	528.98	38.45
O2	530.91	65.67	530.84	64.30	530.77	61.55

**Table 8:** Deconvoluted parameters from the IFNMR spectra of the prepared LCMO samples.

Peaks	LCMO3		LCMO4		LCMO5	
	Frequency (MHz)	Area (%)	Frequency (MHz)	Area (%)	Frequency (MHz)	Area (%)
*f*1 (Mn^4+^)	367.51	28.72	376.83	37.88	377.27	49.74
*f*2 (Mn^3+^)	380.78	44.74	386.21	41.71	387.93	44.59
*f*3 (Mn^3+^)	391.31	26.54	392.24	20.41	397.42	5.67

**Table 9:** Comparison of Mn^4+^/Mn^3+^ valence ratio obtained from XPS and ^55^Mn IFNMR.

Samples	Mn^4+^/Mn^3+^ Valence Ratio		
	XPS	^55^Mn IFNMR	Nominal
LCMO3	0.39	0.40	0.43
LCMO4	0.60	0.61	0.66
LCMO5	0.94	0.99	1.00

## Data Availability

Data will be made available upon request.

## Supplementary Materials

The following supporting information can be downloaded at: https://www.mdpi.com/article/10.3390/molecules31173122/s1, Figure S1: Rietveld refined graphs of the prepared LCMO3, LCMO4 and LCMO5 samples. The red circles represent the experimentally observed X-ray diffraction intensities, while the black solid line corresponds to the diffraction pattern calculated from the Rietveld refinement. The blue line at the bottom denotes the difference profile between the observed and calculated intensities, demonstrating the quality of the refinement. The magenta vertical tick marks indicate the Bragg reflection positions corresponding to the crystallographic planes of the refined phase. Figure S2: XPS survey spectra of the prepared LCMO3, LCMO4 and LCMO5 samples. Figure S3: (a–c) Mn 2p core–level spectra and (d–f) O 1s core–level spectra for the LCMO samples.
