# Supplementary material for: Effect of Ca2+ Doping on the Structural, Magnetic and Magneto–Transport Properties of La1−xCaxMnO3 Manganites: Insights from XPS and 55Mn IFNMR Studies
Source: Molecules. 2026 Sep 6;31(17):3122. doi: 10.3390/molecules31173122 (PMC13567049; doi:10.3390/molecules31173122)
Supplement: Supplementary file 1 [file molecules-31-03122-s001.zip › molecules-4384344-supplementary.pdf]

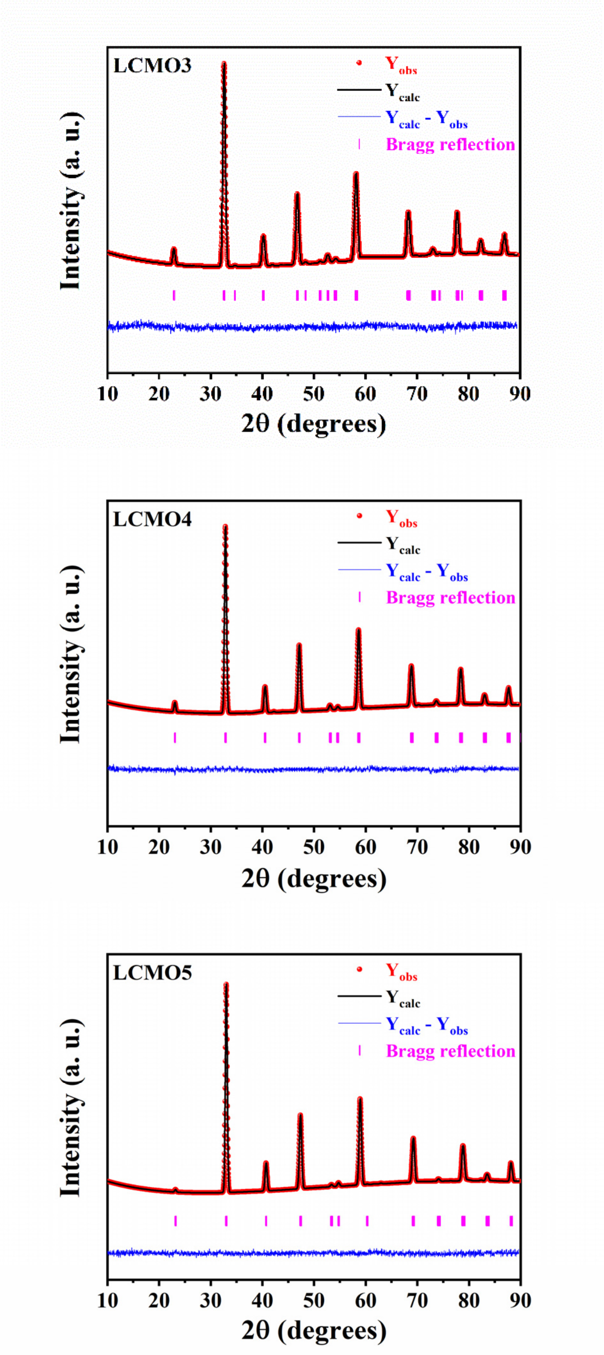

Figure S1. Rietveld refined graphs of the prepared LCMO3, LCMO4 and LCMO5 samples. The red circles represent the experimentally observed X-ray diffraction intensities, while the black solid line corresponds to the diffraction pattern calculated from the Rietveld refinement. The blue line at the bottom denotes the difference profile between the observed and calculated intensities, demonstrating the quality of the refinement. The magenta vertical tick marks indicate the Bragg reflection positions corresponding to the crystallographic planes of the refined phase.

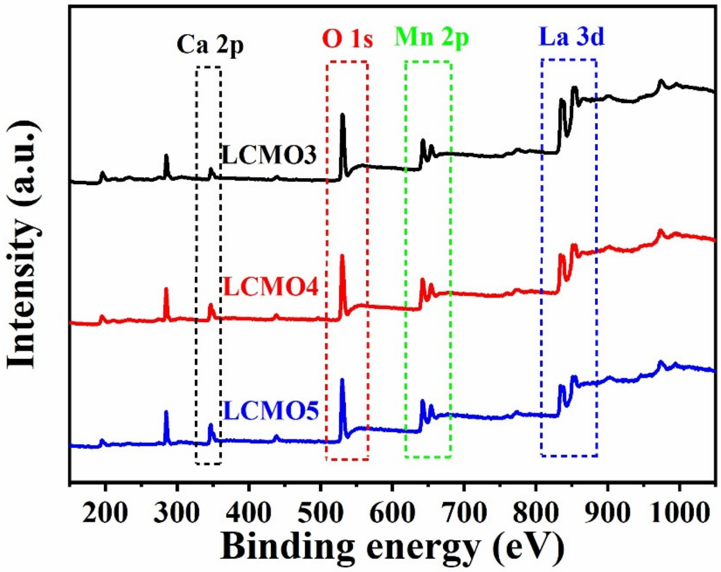

Figure S2. XPS survey spectra of the prepared LCMO3, LCMO4 and LCMO5 samples.

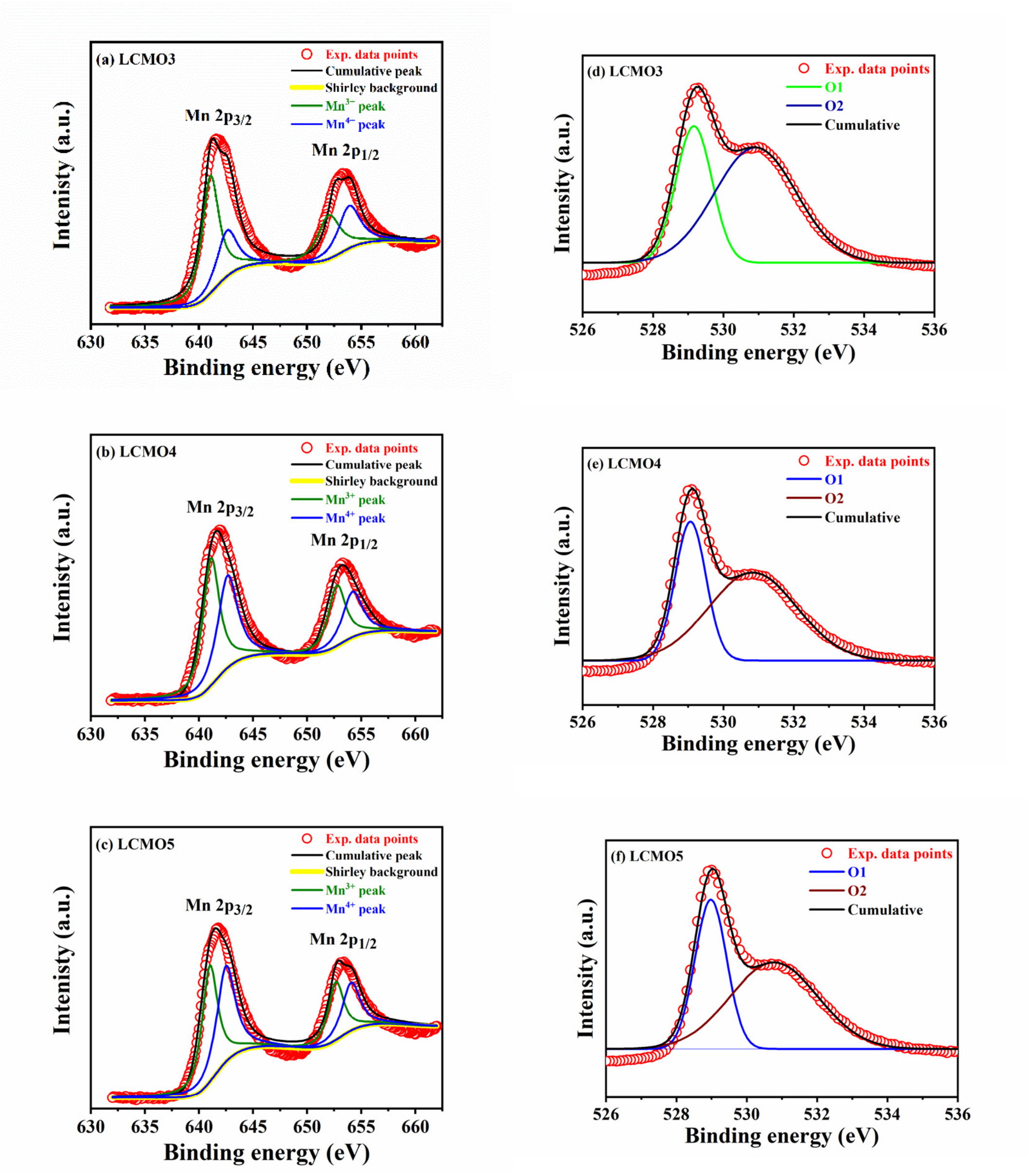

Figure S3. (a – c) Mn 2p core – level spectra and (d – f) O 1s core – level spectra for the LCMO samples.
